# Programmable Bacteria with Dynamic Virulence Modulation System for Precision Antitumor Immunity

**DOI:** 10.1002/advs.202404069

**Published:** 2024-07-26

**Authors:** Leyang Wu, Lin Li, Liyuan Qiao, Chenyang Li, Shuhui Zhang, Xingpeng Yin, Zengzheng Du, Ying Sun, Jiahui Qiu, Xiaoyao Chang, Bohao Wang, Zichun Hua

**Affiliations:** ^1^ Department of Neurology of Nanjing Drum Tower Hospital and The State Key Laboratory of Pharmaceutical Biotechnology School of Life Sciences and The Affiliated Hospital of Nanjing University Medical School Nanjing University Nanjing Jiangsu 21008 P. R. China; ^2^ Nanjing Generecom Biotechnology Co., Ltd. Nanjing Jiangsu 210023 P. R. China; ^3^ Changzhou High‐Tech Research Institute of Nanjing University and Jiangsu TargetPharma Laboratories, Inc. Changzhou Jiangsu 213164 P. R. China; ^4^ Faculty of Pharmaceutical Sciences Xinxiang Medical University Xinxiang Henan 453002 P. R. China

**Keywords:** attenuated salmonella, microbial therapeutics, quorum‐sensing, synthetic biology, tumor bio‐immunotherapy

## Abstract

Engineered bacteria‐mediated antitumor approaches have been proposed as promising immunotherapies for cancer. However, the off‐target bacterial toxicity narrows the therapeutic window. Living microbes will benefit from their controllable immunogenicity within tumors for safer antitumor applications. In this study, a genetically encoded microbial activation strategy is reported that uses tunable and dynamic expression of surface extracellular polysaccharides to improve bacterial biocompatibility while retaining therapeutic efficacy. Based on screening of genes associated with *Salmonella* survival in macrophages, a novel attenuated *Salmonella* chassis strain AIS (*htrA* gene‐deficient) highly enriched in tumors after administration and rapidly cleared from normal organs are reported. Subsequently, an engineered bacterial strain, AISI‐H, is constructed based on the AIS strain and an optimized quorum‐sensing regulatory system. The AISI‐H strain can achieve recovery of dynamic tumor‐specific bacterial virulence through a novel HTRA‐RCSA axis‐based and quorum‐sensing synthetic gene circuit‐mediated increase in extracellular polysaccharide content. These strains act “off” in normal organs to avoid unwanted immune activation and “on” in tumors for precise tumor suppression in mice. The AISI‐H strain shows significant tumor inhibition and potent activation of anticancer immunity in a melanoma mouse model. The AISI‐H strain exhibits excellent biocompatibility. This bacterial regulation strategy expands the applications of microbe‐based antitumor therapeutics.

## Graphical abstract

A novel AISI‐H strain using a screened attenuated *Salmonella* (AIS), which is enriched in tumors and rapidly cleared from normal organs is developed. This strain incorporates a quorum‐sensing system to enhance bacterial biocompatibility through controlled expression of surface polysaccharides while maintaining its antitumor efficacy. The AISI‐H strain achieves bacterial virulence through an HTRA‐RCSA axis‐based increase in extracellular polysaccharide content.

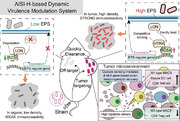

## 1 Introduction

Bacteria‐mediated anticancer therapies have transformed the landscape of tumor immunotherapy. These bacteria preferentially accumulate within tumors, actively penetrate tumor tissue^[^ ^1^ ^]^ can be genetically modified to produce anticancer agents in situ.^[^ ^2^ ^]^ Although bacteria‐based tumor therapy shows tremendous potential for biomedical applications, the associated side effects restrict the permissible maximum dose, hindering the attainment of desired outcomes within the therapeutic window and ultimately leading to the termination of several clinical trials.^[^ ^3^ ^]^ Additionally, precise control of the dynamic characteristics of bacteria is required to control their pharmacokinetics in vivo, which is crucial for optimizing treatment effectiveness and reducing adverse reactions. Off‐target effects of bacteria outside tumors, pathogenic stimulation, and premature release of loaded drugs from strains may cause healthy tissue damage.^[^ ^4^ ^]^ Therefore, the safety of bacteria‐based biotherapeutics should not be ignored.

One of the key reasons for bacterial toxicity is the prolonged survival and immune activation in healthy organs. For instance, although showing promising preclinical antitumor effects, attenuated *Salmonella typhimurium* VNP20009 remains detectable in healthy organs for extended periods after administration.^[^ ^4^, ^5^ ^]^ These strains can persist within macrophages by generating *Salmonella*‐containing vacuoles (SCVs) are accompanied by a series of biological stresses.^[^ ^6^ ^]^ Intracellularly replicating bacteria can trigger macrophage oncosis, followed by a massive bacterial rerelease and immunostimulation, ultimately triggering a prolonged toxic response in the body.^[^ ^6^, ^7^ ^]^ Experiments involving crab‐eating monkeys have indicated that *Salmonella* can persist in normal organs for more than 40 days.^[^ ^5^ ^]^ Therefore, the biosafety of bacterial therapies could theoretically be enhanced if the survival and immunogenicity of *Salmonella* in healthy organs could be controllably reduced.

Knocking out genes that produce bacterial immunogenic antigens, such as extracellular polysaccharides (EPS)^[^ ^1^, ^5^, ^8^ ^]^ and flagellin,^[^ ^9^ ^]^ or those that affect bacterial metabolism,^[^ ^10^ ^]^ is an effective way to circumvent bacterial biotoxicity. However, this strategy has difficulty achieving an optimal balance between efficacy and safety and is often accompanied by permanent strain attenuation and decreased intratumoral colonization, as observed in clinical trials.^[^ ^3^, ^11^ ^]^ Non‐systemic in situ intratumor injection avoids this problem; however, this approach is far from the best drug administration method.^[^ ^2^, ^12^ ^]^ An emerging focus in synthetic biology is the modification of microorganisms to enable strain‐specific growth or protein expression in situ in response to conditions such as population density,^[^ ^13^ ^]^ oxygen,^[^ ^14^ ^]^ and small molecule compounds.^[^ ^13b^ ^]^ Thus, designing genetic circuits to activate bacterial immunogenicity within tumors to accommodate biosafety and therapeutic efficacy potentially addresses clinical translation issues for microbial therapeutics in cancer treatment.

Here, we propose a novel tumor‐specific bacterial immunogenicity restoration‐based microbial therapy that combines an optimized quorum‐sensing regulatory system and a screened novel attenuated *Salmonella* chassis strain for precise antitumor treatment. Specifically, when compared with the VNP20009 strain, an attenuated *Salmonella* Δ*htrA*‐VNP (named AIS) strain with substantially reduced biotoxicity while retaining high intratumor enrichment was obtained through bacterial transcriptome analysis and CRISPR screening. The chassis strain was integrated with a quorum‐sensing system that regulates protein expression to gain a newly engineered bacterium, AISI‐H, which allows tumor‐specific bacterial immunogenicity recovery based on HTRA protein reexpression. This design prioritizes safety while preserving robust anticancer efficacy, demonstrating its promise for potential biomedical applications.

## 2 Results

### 2.1 CRISPR‐Based Knockout Screen Reveals *htrA* As a Key Regulator of *Salmonella*‐Mediated Biotoxicity

**Figure 1:**
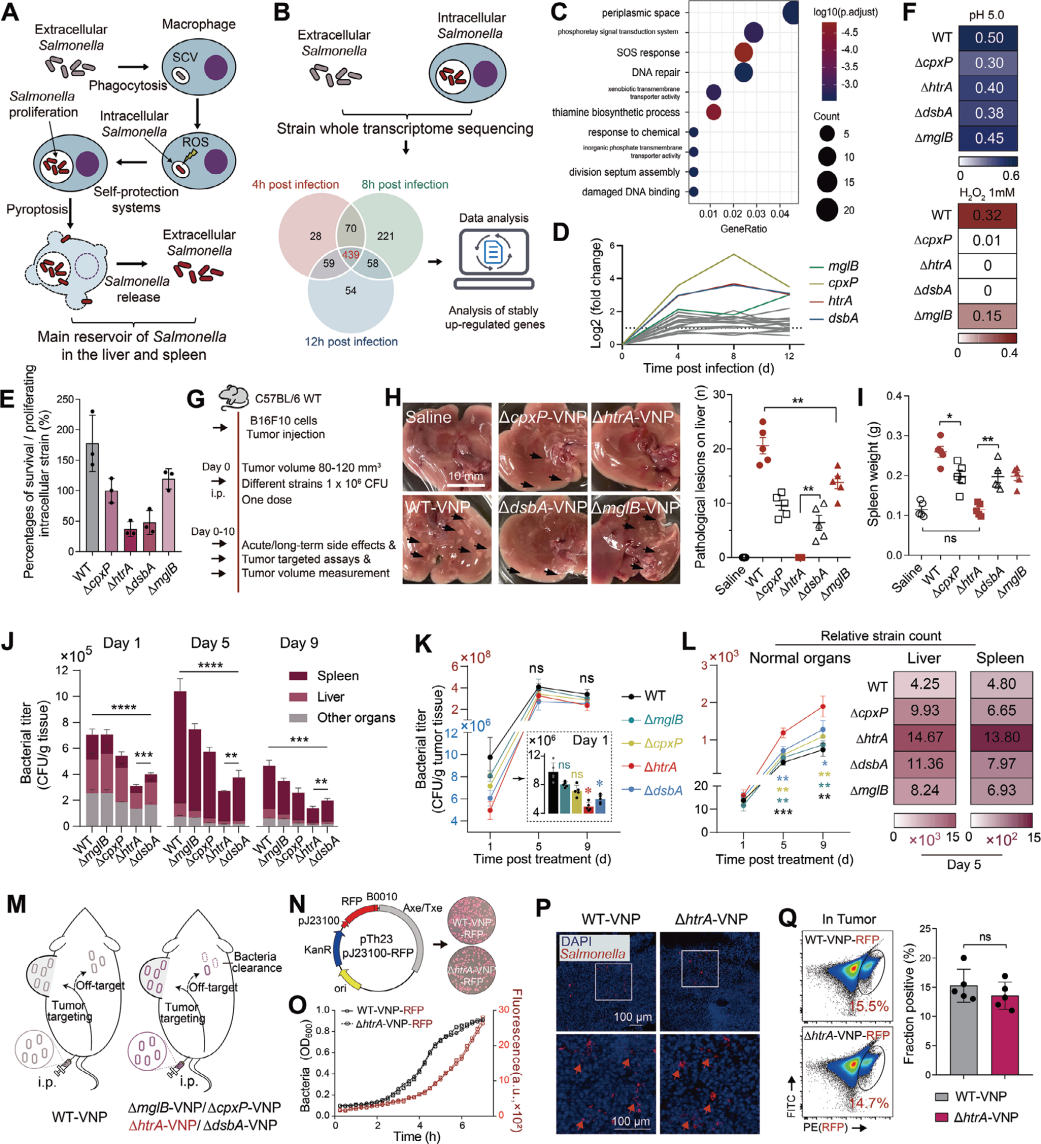
Attenuated *Salmonella* Δ*htrA*‐VNP demonstrates superior biosafety and tumor‐specific enrichment. A) Schematic of *Salmonella* escaping macrophage killing. Intracellular *Salmonella* avoid being cleared by macrophages through the production of Salmonella‐containing vacuole (SCV) and a series of metabolic behaviors. Intracellular strains can continue to proliferate and be released by inducing macrophage oncosis. B) Bacterial transcriptome data were analyzed for *Salmonella* before entry into macrophages (0 h) and 4, 8, and 12 h after entry into cells. Venn diagram shows number of genes upregulated in the bacterial genome at 4/8/12 h compared to 0 h. A total of 439 genes were found to be simultaneously upregulated at multiple time points (4/8/12 h). C) GO analysis of commonly upregulated genes in (B). D) Changes in upregulated periplasmic space‐related genes in (C) at 4, 8, and 12 h compared to 0 h. E) Comparison of the survival/proliferation ability of different attenuated *Salmonella* in macrophages (*n* = 3). F) Comparison of the OD_600_ values of different attenuated *Salmonella* after 4 h of incubation in medium at pH 5.0 (top) or supplemented with 1 mM hydrogen peroxide (bottom). The means are displayed (*n* = 3). G) Schematic of the biosafety and intratumor enrichment assays of different attenuated *Salmonella* in B16‐F10 tumor‐bearing mice. H) Comparison of acute liver injury in each group of mice after 1 day of treatment. Representative images of liver injury in each group (left) and statistical comparisons of the liver lesions number (right) are shown (*n* = 5). The black arrow points to the liver injury site. I) Comparison of spleen weights in each group of mice after 1 day of different treatments (*n* = 5). J) Comparison of bacterial titers in the spleen, liver, and other normal organs (including the heart, lung, and kidney) in mice after 1, 5, or 9 days of different treatments (*n* = 5). K) Curves of intratumoral bacterial titers in mice after 1, 5, or 9 days of different treatments. The enlarged image shows the comparison of bacterial titers on day 1 (*n* = 5). L) The ratio of bacterial CFUs in tumors to normal organs (including heart, liver, spleen, lung, and kidney) per gram of tissue on days 1, 5, and 9 (left). The mean values of the tumor:liver and tumor:spleen ratios on day 5 are shown (right). M) Schematic of in vivo changes of distribution between the five *Salmonella* species after administration to tumor‐bearing mice. N) Schematic of the plasmid constitutively expressing RFP (left) with representative coated plates of the engineered WT‐VNP‐RFP and Δ*htrA*‐VNP‐RFP strains (right). O) Growth curves and fluorescent intensity curves of the two engineered strains in (N). P) The frozen sections show the distribution of the introtumoral engineered strains 5 days after administration. DAPI‐stained nuclei (blue), orange arrows pointing to RFP‐expressing strains. Scale bar = 100 µm. Q) Flow cytometry was used to compare the intratumoral levels of the two engineered strains in (P). All error bars represent the s.d. ns, no significance. *P < 0.05; **P < 0.01; ***P < 0.001; ****P < 0.0001. Statistics were calculated using the two‐tailed unpaired Student's *t*‐test with Welch's correction.

Prolonged survival of *Salmonella* inside macrophages contributes to consistent adverse host reactions^[^ ^15^ ^]^ (**Figure** **1**A). The genes upregulated in *Salmonella* after phagocytosis by macrophages were analyzed to identify the key genes responsible for *Salmonella* resistance to macrophage‐mediated killing (Figure 1B). Gene Ontology (GO) analysis revealed significant changes in the periplasmic space‐related genes (Figure 1C), with *cpxP*, *htrA*, *dsbA*, and *mlgB* being the most common (Figure 1D). We speculate that these four genes are directly related to the survival of *Salmonella* in macrophages. In order to test this hypothesis, CRISPR was used to construct corresponding deficient mutants based on VNP20009, a classic attenuated *Salmonella* strain used in clinical antitumor trials.^[^ ^3^, ^5^ ^]^ These strains were then co‐cultured with macrophages. The wild‐type VNP (WT‐VNP) strain survived effectively within macrophages (Figure 1E), in line with previous reports.^[^ ^4a^ ^]^ All four gene‐deficient strains exhibited a substantial decrease in intramacrophage survival, with the most significant reduction occurring in the Δ*htrA*‐VNP strain (79.2% lower than that in the WT‐VNP strain) (Figure 1E). Macrophages rely on acidic (H+) and reactive oxygen species (ROS) to eliminate intracellular bacteria^[^ ^6^, ^16^ ^]^ (Figure S1A, Supporting Information). Consistent with their reduced intra‐macrophage survival, these deficient strains displayed decreased resistance to acidity and oxidative stress (Figure 1F; Figure S1B–D, Supporting Information).

These strains were subjected to in vivo toxicity tests. They were administered intraperitoneally to melanoma‐bearing mice, a tumor model commonly used for bacterial‐mediated antitumor research^[^ ^2a^ ^]^ (Figure 1G). After administration, some strains inevitably act off‐target in normal organs, especially the liver and spleen, causing adverse effects.^[^ ^4^, ^10^ ^]^ Our results showed that WT‐VNP triggered rapid acute hepatic and splenic toxicity, as evidenced by liver damage, splenomegaly, and the onset of sudden weight loss (Figure 1H,I; Figure S2B, Supporting Information). All four defective strains achieved significant toxicity remission, with Δ*htrA*‐VNP exhibiting the greatest reduction (Figure 1H,I; Figure S2B,D,E, Supporting Information). Next, the biodistribution of these strains was examined. All four deficient strains showed significantly lower titers in normal organs than the WT‐VNP strains (Figure 1J). Living bacteria enable tumor‐specific enrichment and proliferation.^[^ ^2a^ ^]^ All five strains showed similarly high intratumor titers (∼10^8^ CFU/g tumor tissue) on day 5 after administration (Figure 1K). The intratumor titers of the four deficient strains were lower than that of the WT‐VNP strain on the first day, with the Δ*htrA*‐VNP and Δ*dsbA*‐VNP strains being more pronounced (Figure 1K). These findings suggest that gene deficiencies affect the initial tumor enrichment of the strains but do not affect their rapid proliferation. However, the antitumor effects of these deficient strains were weakened (Figure S2A,C, Supporting Information). All four deficient strains showed varying degrees of significantly elevated tumor targeting. Compared to that of WT‐VNP, the tumor targeting level (tumor: normal organs) of the deficient strains on day 5 was elevated by 1.31‐fold (Δ*cpxP*‐VNP), 3.00‐fold (Δ*htrA*‐VNP), 1.79‐fold (Δ*dsbA*‐VNP), and 1.51‐fold (Δ*mlgB*‐VNP) (Figure 1L).

These new genetically defective strains achieved an improved balance between high biosafety and a sustained high titer within tumors, and the top‐performing *htrA*‐deficient strain was selected for further investigation (Figure 1M). No differences were significant in the in vitro growth or fluorescence intensity of the two engineered bacteria (WT‐VNP‐RFP and Δ*htrA*‐VNP‐RFP) loaded with a plasmid expressing red fluorescent protein (RFP) (Figure 1N,O). RFP‐expressing strains were observed inside the tumors with high enrichment after administration (Figure 1P,Q). These results suggest that Δ*htrA*‐VNP (named AIS) can be used as a chassis strain similar to the WT‐VNP strain, as the former has more biocompatibility and safety and may be a more promising antitumor biologic.

### 2.2 The AIS Strain Exhibits a Low EPS Content Accompanied by Poor Immunogenicity and Weak Survival in Macrophages

**Figure 2:**
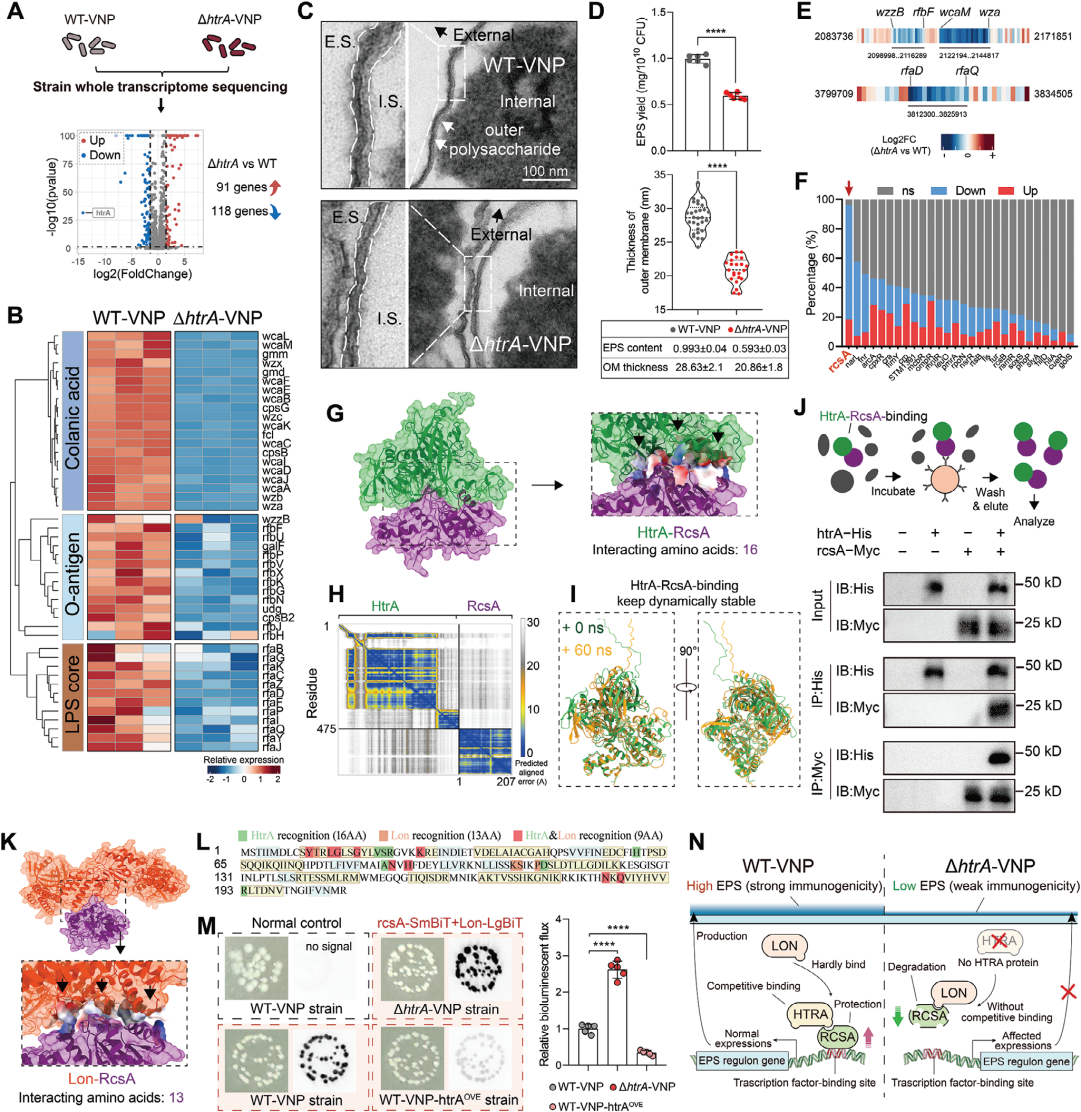
The strain ∆*htrA*‐VNP (AIS) lacks HTRA protection against the transcription factor RCSA, resulting in bacterial extracellular polysaccharide content reduction. A) Volcano plot illustrating the gene expression changes of VNP and AIS strains. B) The expression of partial marker genes for extracellular polysaccharide (EPS), including lipopolysaccharide, O‐glycogen, and kojic acid. C) Representative TEM images of ruthenium red‐stained bacteria showing the EPS on the outer surface of the cell. The *htrA* gene deletion trigger a reduction in the EPS content of strains. The white arrows point to the outer surface of the bacteria. E.S., external space; I.S., internal space. Scale bar = 100 nm. D) Comparison of EPS content at the same bacterial count for different strains (top, *n* = 6). Violin plot reveals change in the outer membrane (OM) thickness of the different strains in (C) (bottom). E) Compared to those in the wild‐type VNP, the changes in gene expression in the Δ*htrA*‐VNP strain were mapped to the chromosome to determine the spatial relationships of coregulated genes in the genome. F) Compared to that in the WT‐VNP, the expression of the genes regulated by major transcription factors in the Δ*htrA*‐VNP strain was altered. G) A novel PPI from the AlphaFold2 prediction dataset shows the interaction between HTRA with RCSA. The zoomed‐in view on the right shows the amino acid sites where the two proteins interact. H) The predicted aligned error (PAE) matrix plot displays a very low predicted alignment error in the relative positions of the HTRA and RCSA. I) The protein conformation at the moment when the RMSD stabilized (60 ns, depicted in light orange) and the initial conformation (0 ns, depicted in light green) were structurally aligned using ChimeraX. A high degree of structural similarity, with an RMSD of 1.035 angstroms between 204 pruned atom pairs (across all 682 pairs: 8.181), was shown. J) A coimmunoprecipitation (co‐IP) experiment was used to detect the interaction between HTRA and RCSA. K) A novel PPI from the AlphaFold2 prediction dataset shows the interaction of LON with RCSA. The zoomed‐in view on the bottom shows the amino acid sites where the two proteins interact. L) Schematic of the amino acid locations predicted by the proteins HTRA and LON to bind to the protein RCSA. M) A NanoBiT tag system was used to validate that HTRA interfered with the interaction between RCSA and LON. Fusions of RCSA and LON to the SmBiT and LgBiT tags were created and tested for interaction in the WT‐VNP, ∆*htrA*‐VNP and WT‐VNP with HTRA overexpression (WT‐VNP‐htrA^OVE^ strains), respectively. Colonies turn dark as a result of protein interactions, and the depth of dark at the same exposure time correlates with the degree of protein interaction. Representative colonies (left) as well as quantitative bioluminescence flux in liquid media (right) are shown (*n* = 5). N) Schematic of the regulation of EPS content by HTRA protein in the strain. HTRA can compete with LON for binding to the RCSA protein, improve RCSA stability, and maintain the stability of RCSA downstream genes, resulting in a high EPS content in the WT‐VNP strain. In the *htrA*‐deficient strain, rapid binding and degradation of RCSA by LON result in the disruption of the expression of downstream genes regulated by RCSA and bacterial EPS content decrease. All error bars represent the s.d. ****P < 0.0001. Statistics were calculated using the two‐tailed unpaired Student's *t*‐test with Welch's correction.

RNA‐seq was conducted to further explore the reasons for AIS strain‐enhanced biosafety, with the VNP strain serving as a control. Hundreds of genes were significantly dysregulated in the *htrA*‐deficient strain (**Figure** **2**A). Several gene clusters associated with the production of extracellular polysaccharides (EPS), including lipopolysaccharide, O‐glycogen, and colanic acid, were downregulated in the AIS strain compared to those in the VNP strain (Figure 2B), which strongly suggested that the former had a lower EPS content. The results of the EPS measurements revealed that the AIS strain had 40.3% lower EPS content than the VNP strain, and transmission electron microscopy indicated a 27.1% reduction in the thickness of the outer membrane with a polysaccharide coating in the AIS strain (Figure 2C,D). These findings demonstrate that defects in the *htrA* gene led to a reduction in the *Salmonella* EPS content. Since EPS protects strains against acidic or oxidative stress,^[^ ^8^, ^17^ ^]^ these are often created by macrophages for intracellular bacterial elimination. Thus, the decreased survival of the AIS strain in macrophages is expected (Figure 1E).

Environmental changes, gene mutations, and other factors typically affect transcript profiles by perturbing transcription factors.^[^ ^18^ ^]^ The *htrA* gene deletion resulted in the simultaneous downregulation of bacterial EPS production and transport‐related gene cluster expression (Figure 2E), which were regulated by the same transcription factor, RCSA.^[^ ^19^ ^]^ Moreover, the RCSA protein also exhibited the most significant influence on all genes whose expression was altered in the AIS strain (Figure 2F; Figure S3A,B, Supporting Information). These results suggest that the HTRA protein may indirectly alter the expression of these genes via the protein RCSA. To explore the interactions between the two proteins, AlphaFold2 was used to predict their tertiary structures and structural relationships. The predictions revealed a strong interaction involving up to 16 amino acids, with a low predicted alignment error between HTRA and RCSA (Figure 2G,H) and a stable binding (Figure 2I; Figure S3C, Supporting Information). This interaction was further confirmed through coimmunoprecipitation (co‐IP) and NanoBiT assays (Figure 2J; Figure S3D, Supporting Information). Furthermore, RCSA overexpression in the AIS strain partially rescued the decrease in the EPS content caused by *htrA* deficiency (Figure S3E, Supporting Information).

The RCSA protein in bacteria can be directly recognized and degraded by the LON protease^[^ ^19^ ^]^ and is involved in recognizing DNA sequences located near multiple gene promoters to regulate downstream EPS‐related gene expression (LON‐RCSA‐EPS axis).^[^ ^19^, ^20^ ^]^ AlphaFold2‐based predictions also indicated an interaction between the LON‐RCSA proteins (Figure 2K). In total, 16 binding sites (green) in the RCSA protein interacted with the LON protease, and 13 binding sites (orange) interacted with the HTRA protein, including nine overlapping competitive binding sites (red), suggesting extremely high binding competition (Figure 2L). Thus, we hypothesized that HTRA could slow RCSA degradation by competitively inhibiting the binding of LON to RCSA, ultimately influencing RCSA‐controlled gene expression. Significant fluorescence signals were observed following the overexpression of Lon‐LgBiT with rcsA‐SmBiT in the WT‐VNP strain, indicating protein‐protein interactions (Figure 2M). This bioluminescence signal was amplified in the AIS strain but was weak in the VNP‐htrA^OVE^ (HTRA overexpression) strain (Figure 2M). These findings indicated that HTRA efficiently prevented LON from binding to RCSA. Moreover, the relative EPS content was considerably greater when the *Lon* gene was deficient in the AIS strain (Δ*htrA&Lon*‐VNP) than in the WT‐VNP strain (Δ*Lon*‐VNP), which further confirmed the involvement of the LON protein in HTRA‐mediated EPS alterations (Figure S3F, Supporting Information).

The AIS strain, with a significantly lower EPS content, showed potentially reduced immunogenicity compared to the VNP strain.^[^ ^17^ ^]^ Consistent with this hypothesis, the AIS strain demonstrated a markedly decreased stimulatory capacity for both macrophages and neutrophils (Figure S4A–C, Supporting Information). Moreover, the AIS strain (0.2%) was much less capable of neutrophil activation in peripheral blood than the VNP strain (2.5%) (Figure S4D, Supporting Information). In vivo, activated neutrophils rapidly remove free strains.^[^ ^21^ ^]^ Therefore, the AIS strain initially had a greater strain titer in the peripheral blood than the VNP strain (Figure S4E, Supporting Information). Over time, the AIS strains in peripheral blood were more swiftly eliminated owing to their low intracellular viability (Figure S4E, Supporting Information). Considering the small amount of residual bacteria, mainly in the spleen and liver, we examined the levels of IL6 and IL1β, classical indicators of systemic toxic cytokines^[^ ^22^ ^]^ in those body parts. Compared to the saline‐treated group, the IL6 and IL1β levels in the spleen and liver of mice significantly increased after VNP‐NC treatment, whereas no significant difference was observed with the AIS strain (Figure S4F,G, Supporting Information). This confirms the greater biocompatibility of the AIS strain. Overall, the enhanced biosafety of the AIS strain can be attributed to the absence of HTRA, resulting in destabilization and subsequent degradation of the transcription factor RCSA by the LON protease. The reduced RCSA protein, in turn, led to decreased EPS synthesis in the strains, bacterial immunogenicity, and diminished survival in macrophages (Figure 2N).

### 2.3 The AIS Strain Coupled with a Genetically Optimized Quorum Sensing System Achieves Tumor‐Specific Protein Expression

**Figure 3:**
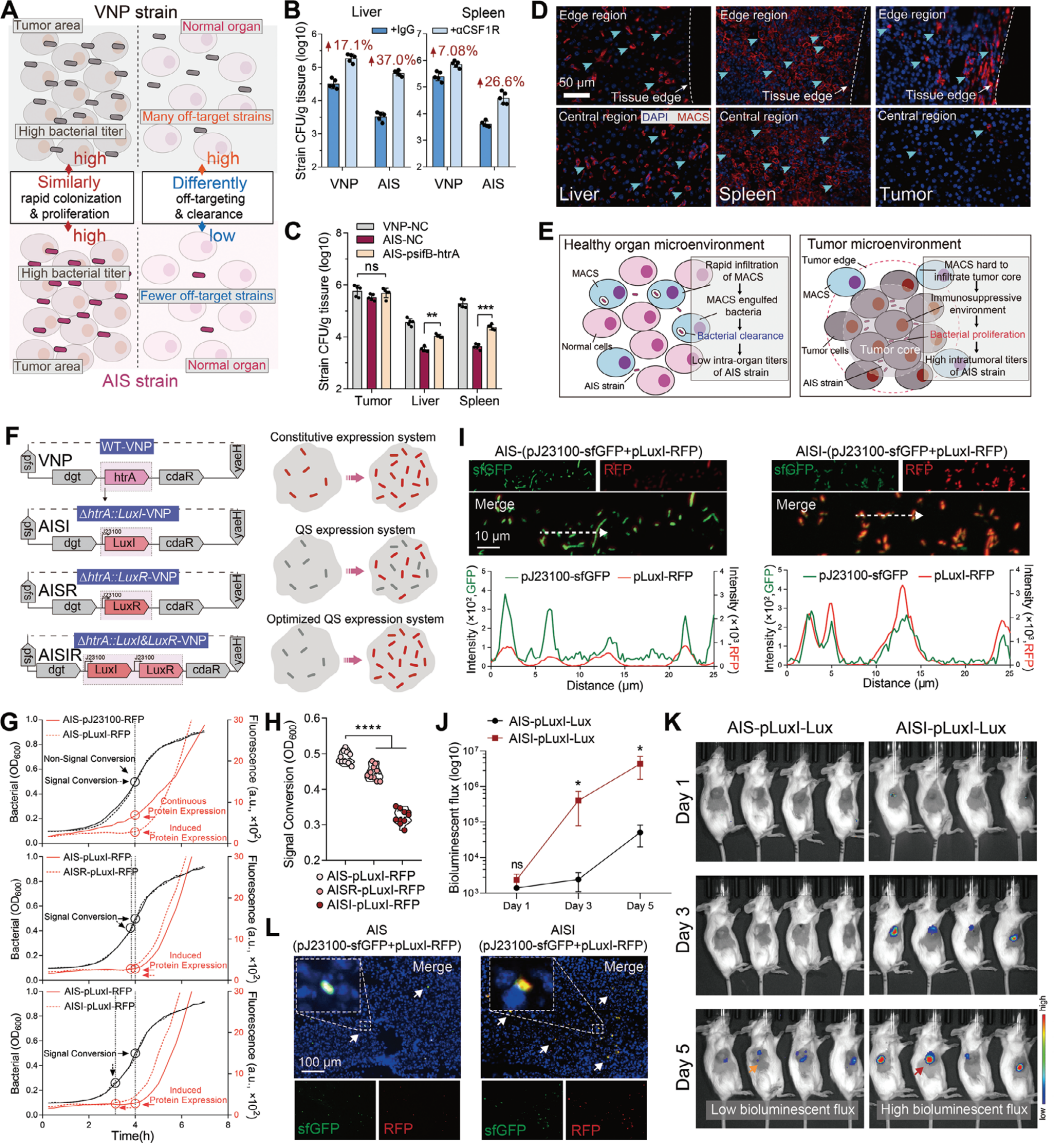
The genetically modified chassis strain ∆*htrA::LuxI*‐VNP (AISI) enables more sensitive QS‐mediated tumor‐specific protein expression. A) Schematic of the distribution of two attenuated *Salmonella* (VNP and AIS) in vivo. B) After depletion of macrophages in vivo, the titers of the VNP and AIS strains in the liver (left) and spleen (right) were compared on day 3 (*n* = 5). C) Comparison of the bacterial titers of three attenuated *Salmonella* (VNP‐NC, AIS‐NC and AIS‐psifB‐htrA) in the tumor, liver, and spleen on day 3 after administration (*n* = 5). D) Immunofluorescence analysis of macrophages distribution in the marginal (top) and core (bottom) regions within the liver, spleen, and tumors of B16‐F10 tumor‐bearing mice. DAPI‐stained nuclei (blue), F4/80‐stained macrophages (red), light blue arrows pointing to macrophages, and dotted lines are the margins of the tissues. Scale bar = 50 µm. E) Schematic of the distribution of macrophages and attenuated *Salmonella* in healthy and tumor tissues. Within healthy tissue, macrophages are evenly distributed, and *Salmonella* can be rapidly recognized and phagocytosed by macrophages. There are fewer macrophages in the core region of the tumor tissue. Thus, little macrophage recognition and phagocytosis of *Salmonella* occurs there, and the bacteria can reach high titers. F) Schematic of three novel genetically modified attenuated *Salmonella* strains generated from VNP (left). The novel strains with the insertion of QS‐related key elements are theoretically more efficient at facilitating QS initiation (right). G) Real‐time growth curves and fluorescent protein expression of strains, including AIS‐pJ23100‐RFP *V.S*. AIS‐pLuxI‐RFP (top), AIS‐pLuxI‐RFP *V.S*. AISR‐pLuxI‐RFP (middle), and AIS‐pLuxI‐RFP *V.S*. AISI‐pLuxI‐RFP (bottom). H) Comparison of the OD_600_ corresponding to the start point of RFP expression for the three genetically modified strains in (G). I) Fluorescence images (top) and quantitative comparison (bottom) of the AIS and AISI strains with both constitutive‐based sfGFP expression and QS‐based RFP expression at the same bacterial density in vitro. Scale bar = 10 µm. J) Changes in the bacterial density and bioluminescence flux in tumors 1, 3, and 5 days after administration (*n* = 5). K) Representative animal images in (J). L) Immunofluorescence of the two engineered strains in tumors with QS‐based RFP and constitutive sfGFP expression. Scale bar = 100 µm. All error bars represent the s.d. ns, no significance. *P < 0.05; **P < 0.01; ****P < 0.0001. Statistics were calculated using the two‐tailed unpaired Student's *t*‐test with Welch's correction.

AIS strain titers in healthy organs were noticeably lower than those of the VNP strain. One speculation is that the macrophages in these organs cleared the AIS strain more efficiently because of decreased intra‐macrophage survival (Figures 1E,**3**A). The macrophages were depleted in vivo using macrophage‐neutralizing antibodies to confirm this (Figure S5A, Supporting Information). The VNP strain titers were significantly upregulated in healthy organs after macrophage depletion (17.1% in the liver and 7.1% in the spleen), whereas the AIS strain was significantly upregulated (37.0% in the liver and 26.6% in the spleen) (Figure 3B). This finding confirmed that macrophage‐mediated AIS clearance contributes to the low titer of the strain in normal organs. For further validation, an intracellular inducible HTRA‐re‐expressing engineered bacteria,^[^ ^23^ ^]^ AIS‐psifB‐htrA, was constructed (Figure S5B–D, Supporting Information). Specific HTRA re‐expression rescued the EPS content and viability of the AIS‐psifB‐htrA strain in macrophages (Figure S5E,F, Supporting Information). Compared with those in the AIS group, the bacterial titer in the AIS‐psifB‐htrA group was significantly greater in normal organs (Figure 3C), and side effects reappeared (Figure S5G,H, Supporting Information). The inner tumoral regions, where the bacteria preferred to reside and lacked sufficient macrophages^[^ ^24^ ^]^ (Figure 3D,E; Figure S5I,J, Supporting Information), coupled with the similar proliferation rates of the AIS and VNP strains, may have contributed to the similarly high intratumoral bacterial titers. However, widely and evenly distributed macrophages in healthy areas (e.g., the liver and spleen) (Figure 3D,E; Figure S5I,J, Supporting Information) led to lower AIS strain titers.

Quorum‐sensing (QS) regulation is a bacterial density‐mediated bioregulatory strategy for target gene expression that has proven successful in bacteria‐based intratumor‐specific drug delivery.^[^ ^13a,c^ ^]^ The AIS strain, which has a high intratumoral titer, was well‐suited for QS system implementation (Figure 3A). QS system initiation correlated with the level of bacteria‐produced autoinducer (AI) signaling molecules, and LuxI and LuxR were found to be key proteins^[^ ^25^ ^]^ (Figure S6A, Supporting Information). We genetically modified the AIS strain to expedite this initiation. Specifically, the *LuxI* and *LuxR* genes were inserted into the vacant *htrA* gene site in the bacterial genome, and the *∆htrA::LuxI*‐VNP (named AISI) and *∆htrA::LuxR*‐VNP (named AISR) strains were obtained (Figure 3F; Figure S6B–E, Supporting Information). Next, the indicator plasmid containing the LuxI promoter and red fluorescent protein (RFP) genes was transfected into these strains. The AIS‐pLuxI‐RFP strains reached a certain density before RFP expression (Figure 3G), which is consistent with previous reports.^[^ ^13a,c^ ^]^ Both modified strains showed lower thresholds for initiating protein expression (Figure 3G,H). The activation coefficient (OD_600_) for the AIS strain is approximately 0.49, while it is 0.45 and 0.32 for the AISR and AISI strains (Figure 3G,H). Given its heightened sensitivity, the chassis strain AISI was selected for subsequent studies, probably attributed to increased AHL (the product of LuxI) synthesis (Figure S6F,G, Supporting Information). Fluorescence imaging demonstrated that RFP expression was higher in the AISI‐pLuxI‐RFP strain than that found in the AIS‐pLuxI‐RFP strain at the same density (Figure 3I).

Strains carrying the LuxI promoter and luciferase operon LuxCDABE were used to evaluate the efficiency of this novel QS system in vivo. The engineered strains were injected intraperitoneally into H22 tumor‐bearing mice, another classical mouse model for bacteria‐mediated antitumor studies.^[^ ^2a^ ^]^ In vivo imaging clearly revealed LuxCDABE protein expression in the AISI‐pLuxI‐Lux group on day 3; it was weaker in the AIS‐pLuxI‐Lux group on day 3 and moderately enhanced on day 5 (Figure 3J,K). Fluorescence micrographs confirmed that the AISI‐pLuxI‐RFP strain exhibited stronger fluorescence within the tumor, which was consistent with the in vitro results (Figure 3I,L). These results reaffirm the superior QS initiation efficiency of the AISI strain compared to that of the AIS strain and contribute to increased target protein production. Compared to the constitutive expression system mediated by the J23100 promoter, the QS‐based expression strategy in the AISI‐pLuxI‐Lux or AISI‐pLuxI‐RFP strains resulted in almost undetectable bioluminescent or RFP signals in normal organs following systemic injection (Figure S7A–G, Supporting Information). This is likely because bacterial titers in normal organs do not reach the threshold required for QS activation. The results of the in vitro fluorescent protein intensity dynamics of the AISI‐pLuxI‐RFP strain imply that this QS‐dependent expression system can ensure that a small number of activated strains detached from high‐density environments will revert to a silent state at low‐density sites (Figure S7H,I, Supporting Information). This QS system, with its specific initiation, can effectively mitigate the toxic side effects of off‐target proteins.

### 2.4 QS System‐Based Tumor‐Specific HTRA Re‐Expression by the AISI Strain Rescues Bacteria‐Mediated Antitumor Effects

**Figure 4:**
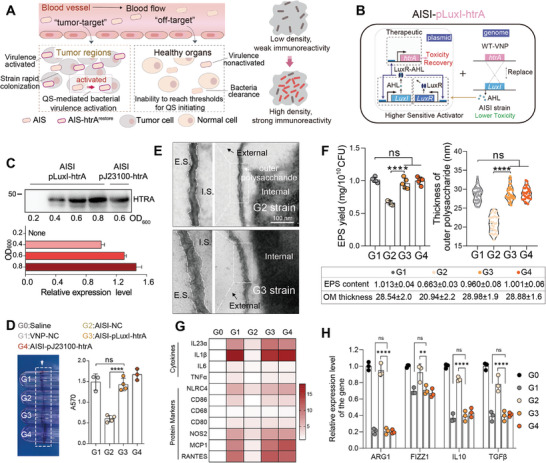
QS‐mediated HTRA‐re‐expression by engineered strain AISI‐pLuxI‐htrA achieves recovery of immunogenicity. A) Schematic of the in vivo distribution of the AISI strain and engineered strain AISI‐pLuxI‐htrA with QS‐mediated HTRA recovery. Both strains are efficiently enriched and proliferate in tumors while few in normal organs. B) Schematic of the controllable virulence recovery circuit based on AISI chassis bacteria. Regulation of *htrA* gene expression by the LuxI promoter to achieve bacterial HTRA re‐expression at high population densities only. C) Immunoblotting analysis determine the relationship between bacterial density and HTRA expression in vitro. D) Representative images of biofilm‐stained test tubes of different strains (left) and quantitative comparisons after biofilm elution (right) are shown. E) Representative TEM images of ruthenium red‐stained strains showing the EPS on the outer membrane of the strain. HTRA‐re‐expression results in a rescued EPS structure on the cell surface of the AISI strain. The white arrows indicate the outer membrane of the bacteria. E.S., external space; I.S., internal space. Scale bar = 100 nm. F) Comparison of EPS content at the same bacterial count for different strains (left, *n* = 6). Violin plot revealing a shift in the outer membrane (OM) thickness of the different engineered strains in (E) (right). G) Detection of antitumor M1‐type macrophage‐related gene expression after coculturing different strains (G0‐G4) with M0‐type macrophages for 6 hours. H) Detection of antitumor M2‐type macrophage‐related gene expression after coculturing different strains (G0‐G4) with M2‐type macrophages for 6 hours. All error bars represent the s.d. ns, no significance. **, *P* < 0.01; ****, *P* < 0.0001. Statistics were calculated using the two‐tailed unpaired Student's *t*‐test with Welch's correction.

Next, an engineered AISI strain combining the quorum‐sensing promoter and the *htrA* gene was constructed (AISI‐pLuxI‐htrA). Theoretically, this strain could restore tumor‐specific immunogenicity (**Figure** **4**A,B). The AISI‐pJ23100‐htrA strain, which constitutively expresses HTRA, was used as a positive control. Western blot analysis confirmed that the HTRA levels increased with increasing strain density (Figure 4C). The biofilm content of the HTRA‐rescued strain did not differ significantly from that of the VNP‐NC strain (Figure 4D). The outer polysaccharide layer thickness and EPS content of the AISI‐pLuxI‐htrA strain were greater than those of the AISI‐NC strain and were not significantly different from those of the VNP‐NC strain (Figure 4E,F). These results indicate that QS system‐based HTRA re‐expression restored the EPS of the AISI strain to a level close to that of the VNP strain. Moreover, the growth profile of the AISI‐pJ23100‐htrA strain was similar to that of the VNP‐NC strain under both acidic and oxidative conditions. The growth of the AISI‐pLuxI‐htrA strain was also similar to that of the VNP‐NC strain because of auto‐inducer accumulation‐induced strain activation over time in a closed culture environment (Figure S8A–E, Supporting Information). Finally, the AISI‐pLuxI‐htrA strain regained intracellular viability close to that of the VNP‐NC strain (Figure S8F, Supporting Information). Compared to the AISI‐NC strain, the AISI‐pLuxI‐htrA strain more significantly promoted the differentiation of M0‐type macrophages into M1‐type macrophages and remodeled M2‐type macrophages (Figure 4G,H). The effective activating effect by the former may derive from the bacterial EPS‐based stimulation via classical TLR4‐NFkB signaling axis on macrophages (Figure S8I, Supporting Information).^[^ ^26^ ^]^ Additionally, this strain enhanced the production of NETs and reactive oxygen species from neutrophils compared to the AISI‐NC strain (Figure 4G; Figure S8G, Supporting Information). Compared with the AISI‐NC strain, the AISI‐pLuxI‐htrA strain induced higher levels of apoptosis in B16F10 cells in vitro, similar to the VNP‐NC strain (Figure S8H, Supporting Information). Collectively, these results indicate that HTRA restoration reversed the stress resistance, immune cell activation capacity, and direct tumor cell‐killing ability of the AISI strain.

**Figure 5:**
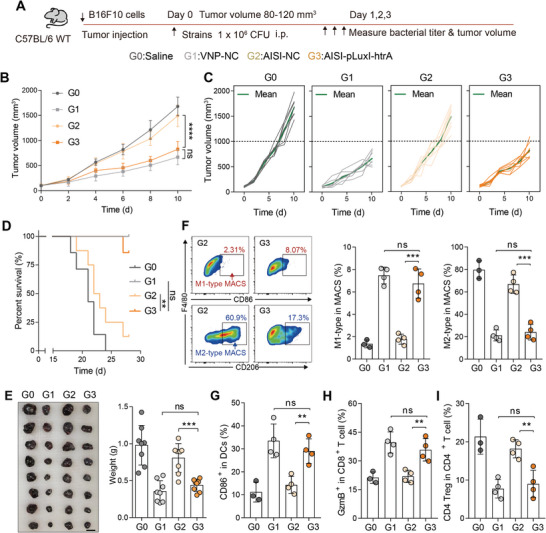
QS‐mediated HTRA‐re‐expression by engineered strain AISI‐pLuxI‐htrA achieves efficient anticancer therapy. A)Schematic of the different strains used for antitumor therapy. B) Tumor growth profiles after different treatments in (A) (*n* = 7). C) Tumor growth curves for each mouse in the different groups in (B). D) Survival curves for the mice from tumor inoculation. The mice were killed when they reached a humane endpoint. E) Tumors were photographed (left) and weighed (right) on day 10 after administration in (B). F) A representative flow cytometric plot (left) and bar chart(right) showing the change in the frequency of M1‐type (F4/80+CD86+) antitumor tumor‐infiltrating macrophages and M2‐type (F4/80+CD206+) pro‐tumor tumor‐infiltrating macrophages in tumors. G) Change in the percentage of mature DCs (CD11c+ MHCII+ CD86+) in tumors. H) Change in the percentage of CD8+ Teffs (CD8+GzmB+) in tumors. I) Change in the percentage of CD4+ Tregs (CD4+Foxp3+) in tumors. *n* = 3 or 4 mice per group in G–I. All error bars represent the s.d. ns, no significance. **P < 0.01; ***P < 0.001; ****P < 0.0001. Statistics were calculated using the two‐tailed unpaired Student's *t*‐test with Welch's correction.

Subsequently, the engineered strain was administered intraperitoneally to B16‐F10 tumor‐bearing mice for anticancer assessment (**Figure** **5**A). The VNP‐NC strain demonstrated superior antitumor effects, which were abolished by the AISI‐NC strain (Figure 5B,C). The HTRA‐rescued AISI‐pLuxI‐htrA strain exhibited robust antitumor efficacy and extended survival time to a level similar to that of the VNP‐NC strain (Figure 5B–E). When compared to the AISI‐NC group, the tumor weight in the AISI‐pLuxI‐htrA group was 45.7% lower at 10 days after administration, and no significant difference was observed in the tumor weight in the VNP‐NC group (Figure 5E). To further investigate the antitumor mechanism of the engineered strain AISI‐pLuxI‐htrA, tumor microenvironmental changes were detected three days after administration in B16‐F10 tumor‐bearing mice (Figure 5A). Enzyme‐linked immunosorbent assays demonstrated that the AISI‐pLuxI‐htrA strain effectively increased the key antitumor cytokines level, including IFNγ and TNFα, within the tumor and peripheral blood compared to those in the AISI‐NC strain (Figure S9A,B, Supporting Information). Flow cytometry assays demonstrated that both the VNP‐NC and AISI‐pLuxI‐htrA strains increased the proportion of antitumor M1‐type tumor‐associated macrophages (TAMs) and decreased the proportion of pro‐tumor M2‐type TAMs (Figure 5F; Figure S9C, Supporting Information). The proportion of M1‐type TAMs in the AISI‐pLuxI‐htrA group was 3.79 times than that in the AISI‐NC group, whereas the proportion of M2‐type TAMs was only 36.0% of that in the latter (Figure 5F). Dendritic cells (DC) are crucial antigen‐presenting cells, and their surface markers, CD80 and CD86, are upregulated during maturation. Compared to the PBS group, significant activation of DC was observed in both the VNP‐NC and AISI‐pLuxI‐htrA treatment groups, whereas the AISI‐NC treatment group showed almost no difference (Figure 5G). This indicated that the restored intratumoral virulence of the AISI‐pLuxI‐htrA strain enhanced DC maturation. The activation of T cells is critical for a particular immunological response. Mature DCs can effectively activate CD8+ T cells.^[^ ^27^ ^]^ A significantly higher proportion of effector CD8+ T cells (Teffs) is observed in the AISI‐pLuxI‐htrA group (Figure 5H). Compared to those in the AISI‐NC group, the proportions of Teffs in the VNP‐NC and AISI‐pLuxI‐htrA groups were 1.81 and 1.63 times greater, respectively (Figure 5H). In addition, the AISI‐pLuxI‐htrA strain reduced the proportion of Treg cells among CD4+ T cells compared to the AISI‐NC strain (Figure 5I). The difference between strains VNP‐NC and AISI‐pLuxI‐htrA was not significant in their ability to upregulate antitumor cytokine and immune cell frequency, and downregulate pro‐tumor immune cell frequency within the tumor (Figure 5F–I; Figure S9, Supporting Information). The novel engineered bacterium AISI‐pLuxI‐htrA, which specifically expresses HTRA in tumors based on an optimized QS system, can robustly activate antitumor immunity by restoring bacterial immunogenicity, which may contribute to its potent anticancer function.

### 2.5 QS System‐Based Tumor‐Specific HTRA Re‐Expression by the AISI Strain Ensures Biosafety and Ability to Translocate to Distal Tumor

**Figure 6:**
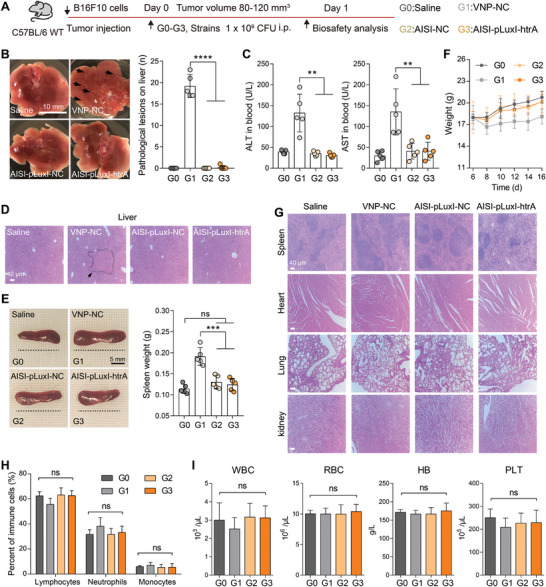
The novel engineered strain AISI‐pLuxI‐htrA obtains high biocompatibility. A) Schematic of the different strains used for biocompatibility analysis. B) Comparison of acute liver injury in each group of mice on day 1 after different administrations. Representative images of liver injury (left) with statistical comparisons of liver lesions number (right) are shown (*n* = 5). C) Blood biochemical analyses of mice with alanine transaminase (ALT) and aspartate aminotransferase (AST) indices. D) Close‐up view of representative H&E staining shows liver injury (black arrow) on day 1 after treatment. Scale bar = 40 µm. E) Comparison of spleen weights 1 day after intraperitoneal administration of saline or an equal dose of the three attenuated *Salmonella* strains (*n* = 5). Scale bar = 5 mm. F) Changes in the body weights of tumor‐bearing mice after different treatments (*n* = 5). G) Close‐up view of representative H&E‐stained spleen, heart, lung and kidney sections. Scale bar = 40 µm. H,I) Comprehensive hematology of blood from tumor‐bearing mice 1 day after administration of different strains. Comparisons of the percentages of immune cells in (H) and white blood cell (WBC) and red blood cell (RBC) counts, hemoglobin (HB) count, and platelet (PLT) count in the blood in (I) are shown (*n* =  5). All error bars represent the s.d. Statistics were calculated using the two‐tailed unpaired Student's *t*‐test with Welch's correction. ns, no significance. ***P < 0.001.

Systemic injection allows bacteria to reach various tumor regions in the body quickly and conveniently. However, this method of administration still poses challenges because the accumulation of strains in normal organs can trigger acute inflammatory reactions. Owing to the lower EPS content of AIS strains, they have a weaker ability to resist clearance by macrophages, making survival in the intact immune surveillance environment of normal organs more challenging (Figure 3A–C), theoretically reducing the risk of adverse effects significantly. Additionally, the titers of the AIS strains in normal organs did not meet the response threshold of the QS system, leading to no initiation of target protein expression in normal organs (Figure S7, Supporting Information). Therefore, the AISI‐pLuxI‐htrA strain may have high biosafety, similar to that of the AIS strains, by avoiding excessive immune activation in healthy tissues. After intraperitoneal administration for one day, the VNP‐NC strains induced significant liver injury (**Figure** **6**A–C), acute splenomegaly (Figure 6D), and a sharp decrease in body weight (Figure 6E) in tumor‐bearing mice. These side effects were significantly alleviated in mice treated with the AISI‐NC or AISI‐pLuxI‐htrA strain (Figure 6A–E). In addition, H&E‐stained sections of the spleen, heart, lungs, and kidneys, as well as routine blood tests, showed that both AIS‐NC‐ and AISI‐pLuxI‐htrA strain‐treated mice performed normally. These data indicate that the AISI‐pLuxI‐htrA strain is a safe antitumor bacterial strain. The safety and antitumor efficacy of the AISI‐pLuxI‐htrA strain were also validated in different tumor models (Figure S10A, Supporting Information). In a mouse subcutaneous H22 hepatocellular carcinoma model and a mouse subcutaneous A20 B‐cell lymphoma model, the tumor weight in the AISI‐pLuxI‐htrA‐treated group was 40.9% and 48.4% lower, respectively, on day 10 post‐treatment than that in the AISI‐NC‐treated group (Figure S10B,C,G,H, Supporting Information). The AISI‐pLuxI‐htrA strain in both models demonstrated safety comparable to that of the AISI‐NC strain, avoiding rapid weight loss during treatment and not causing acute spleen or liver toxicity, both of which were commonly accompanied by the wild‐type VNP strains (Figure S10D–F, I–K, Supporting Information). These data indicate that the AISI‐pLuxI‐htrA strain is a safe antitumor bacterial strain.

**Figure 7:**
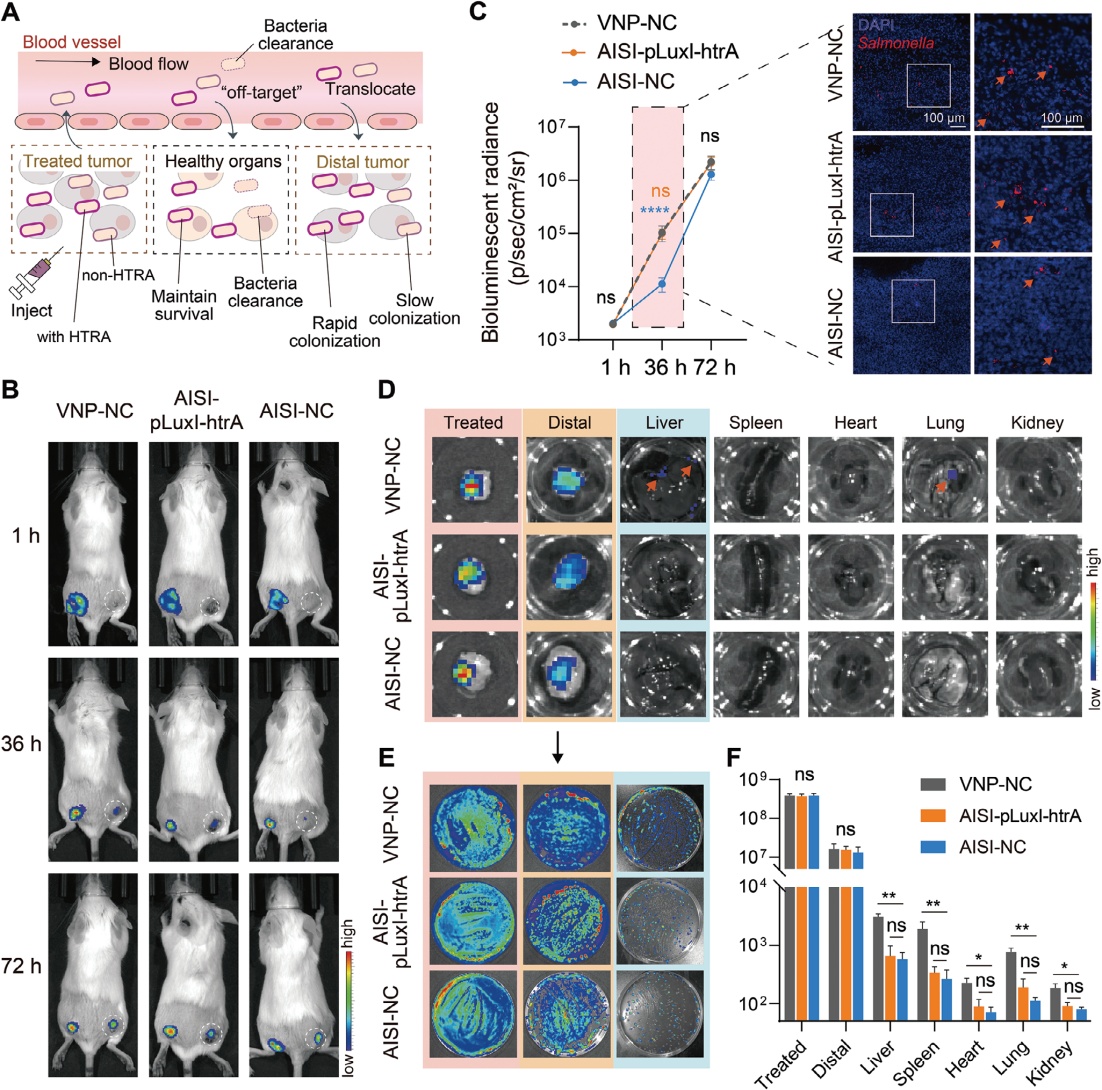
The novel engineered strain AISI‐pLuxI‐htrA enables precisely and efficiently translocation to distal tumors. A) Schematic of HTRA‐mediated bacterial translocation. Attenuated *Salmonella* AISI‐pLuxI‐htrA was injected into a single tumor (treated tumor), and HTRA‐re‐expression accompanied by an increase in extracellular polysaccharide production enabled bacterial translocation to distal tumors more quickly. In the normal region, quorum‐sensing‐off accompanied by HTRA lost and the strain was rapidly removed. B) Translocation of VNP‐NC, AISI‐pLuxI‐htrA and AISI‐NC strains to distal tumors in H22 syngeneic mouse tumor models. The above bacteria were transfected with a plasmid that consistently expressed the biophoto LuxCDABE. Representative IVIS images showing bacterial translocation in vivo at 1, 36, and 72 hours postadministration. The circular dotted line shows the distal tumor site. The black arrows indicate the location of the bacterial injection. The orange arrows indicate the location of bacterial translocation. C) Changes in the bacterial density bioluminescent flux in distal tumors 1, 36, and 72 h after administration (left). A representative cryosection image of fluorescence from distal tumors at 36 hours after administration showing cell nuclei (DAPI, blue) and *Salmonella* (RFP, red) (right). The three strains were transfected with a plasmid that consistently expressed RFP. Orange arrows indicate introtumoral bacteria. Scale bar = 100 µm. D) Representative images of *ex vivo* organs taken with an IVIS showing bacterial tumor translocation at 72 hours in (B). Orange arrows point to bacteria that are off‐target on normal organs. E) Representative images of the bacterial colonies on agar plates after coating the treated tumors, distal tumors, and liver tissue in (D). F) Comparison of bacterial titers in different organs in (E). All error bars represent the s.d. Statistics were calculated using the two‐tailed unpaired Student's *t*‐test with Welch's correction. ns, no significance. *P < 0.05; **P < 0.01; ****P < 0.0001.

Compared to systemic injection, intratumoral injection often has higher safe doses and better therapeutic effects, allowing bacteria to translocate from the initially injected tumor site to deeper and less accessible tumor sites (**Figure** **7**A), enabling the strain to achieve systemic antitumor effects.^[^ ^28^ ^]^ However, off‐target migration of bacteria may pose serious safety issues. Tracking the colonization kinetics with the bioluminescent strain confirmed that the VNP‐NC, AISI‐NC, and AISI‐pLuxI‐htrA strains could effectively translocate to the distal tumor within 36 h after administration to one side of the tumor. The AIS‐pLuxI‐htrA strain exhibited a stronger translocation ability than the AIS‐NC strain, similar to the WT‐VNP strain (Figure 7B,C). This may be attributed to the in situ expression of htrA, which increases the EPS levels of the strain, increases bacterial bioavailability in circulation, and promotes bacterial translocation to distal tumors. Moreover, markedly less strain colonization was detected in healthy organs in the AIS‐pLuxI‐htrA group than in the WT‐VNP group (Figure 7D–F). This difference may be attributed to hindered HTRA expression due to strain titers below the QS system response threshold in normal organs, allowing the AIS‐pLuxI‐htrA strain to revert to attenuated activity and thus be readily cleared from healthy organs. In conclusion, the AISI‐pLuxI‐htrA strain exhibited similar antitumor activity and distal tumor translocation capacity as the VNP‐NC strain, but lower off‐targeting of the translocation process ensured its higher biosafety.

## 3 Discussion

**Scheme 1:**
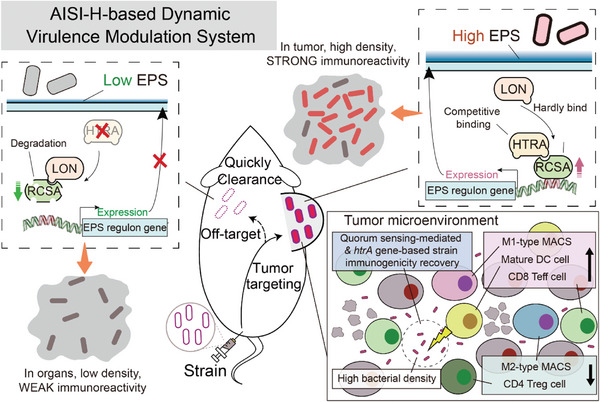
Schematic diagram of the dynamic modulation effect of AISI‐H (AISI‐pLuxI‐htrA) strain virulence. After systemic injection of AISI‐H strain, off‐target strains distributed to normal organs could not trigger the QS system to express HTRA due to low titer, failing to protect RCSA from LON degradation, thereby maintaining attenuating activity and more likely to be cleared. In contrast, rapid proliferation of intra‐tumor strains triggered the QS system to express HTRA, protecting RCSA‐mediated EPS synthesis thereby restoring virulence. Potent anti‐tumor effects were achieved by enhancing the anti‐tumor phenotype, including increasing the levels of anti‐tumor factors and the proportion of anti‐tumor immune cells (mature DC cells, M1‐like macrophages and CD8 Teffs) and decreasing the proportion of pro‐tumor immune cells (M2‐like macrophages and CD4 Tregs).

In this study, we screened a novel attenuated *Salmonella typhimurium* chassis strain, Δ*htrA*‐VNP (named AIS). The strain was found to have reduced immunogenicity and intramacrophage survival owing to a reduction in the extracellular polysaccharide content by affecting the LON‐RCSA‐EPS axis. The AISI strain, which integrates the AIS strain with optimized QS triggers, induces protein expression in high‐density colonies present only in tumors while remaining in non‐tumor areas. We then used the QS trigger system for the tumor‐specific re‐expression of HTRA in the AISI strain (AIS‐pLuxI‐htrA, AISI‐H) to activate bacterial immunogenicity, thus successfully achieving the efficacy and biosafety of bacteria‐mediated antitumor therapy (**Scheme** **1**).

The dose‐limiting toxicity of bacteria‐mediated antitumor therapies is a long‐standing challenge. Although live bacterial mixtures and wild‐type *Salmonella* have been shown to cause tumor infection and regression in humans,^[^ ^29^ ^]^ the potential risk of infection limits the replication of such strategies. Genetically modified attenuated strains offer a solution to this dilemma, albeit with reduced efficacy and in the presence of strain with off‐target toxicity, as shown in clinical trials.^[^ ^3a^ ^]^ In this study, we addressed this issue by knocking out the *htrA* gene in the VNP20009 genome, thereby creating a safer chassis strain (Δ*htrA*‐VNP). Previous studies have reported various functions of the HTRA protein in different bacterial strains, including *Salmonella*, given its role as a classical serine protease.^[^ ^30^ ^]^ For example, it has been demonstrated that deletion of the *htrA* gene leads to increased susceptibility of *Salmonella* to temperature and oxidative stress, decreased virulence in TNFR1‐KO mice, and reduced survival ability in macrophages.^[^ ^31^ ^]^ Although some of these findings align with those of the present study, our research offers several novel contributions. In this study, we have: 1) discovered, for the first time, the role of HTRA in regulating extracellular polysaccharides beyond its classical molecular chaperone/protease functions, as revealed by transcriptomic analysis of *htrA*‐deficient *Salmonella*; 2) described, for the first time, the HTRA‐RCSA‐LON‐based EPS regulatory pathway, which is pivotal for comprehensive understanding and characterization of HTRA function; 3) provided the first immunological explanation wherein *htrA*‐deficient *Salmonella* was rapidly cleared in normal organs but maintained a high titer in tumors; and 4) pioneered the coupling of *htrA*‐deficient *Salmonella* with a quorum‐sensing system for anticancer therapy. The main reasons why the wild‐type bacteria trigger excessive immune activation in healthy tissues are 1) the presence of high titers of off‐target bacteria and 2) strong bacterial immunogenicity, inducing excessive activation of immune cells. The AISI strain constructed here provides evasive strategies, including 1) weakening bacterial viability in healthy tissues without affecting proliferation within tumors and 2) reducing bacterial immunogenicity in healthy tissues but achieving recovery within tumors through a QS system. Ultimately, the AISI strain effectively activated intratumoral immunity without producing excessive immune activation in normal tissues.

Another detoxification strategy involves covering the microbial surface with molecular/biosynthetic compounds for bacterial camouflage to achieve in vivo stealth.^[^ ^32^ ^]^ However, these disposable static modifications to bacteria do not permit in situ modulation and may result in compromised bacterial bioactivity or shedding of surface modifications mediated by natural bacterial growth, thereby reducing efficacy. The QS system integrated with the novel attenuated *Salmonella* AISI can address these limitations by selectively reestablishing bacterial immunogenicity within tumors without unforeseen side effects. This genetic modification can be reliably and consistently sustained, and the AISI strain is unlikely to reach a density that induces its expression in off‐target tissues.

Once bacteria‐mediated drug delivery occurs at the wrong time/place, it can hinder efficient protein binding to pharmacodynamic targets and may even lead to toxic effects.^[^ ^2c^ ^]^ Our findings validate the substantial off‐target nature of bacteria‐driven constitutive protein expression. Therefore, the reprogramming of bacteria to create “bacterial robots” with high tumor‐specific toxicity is crucial for optimizing bacterial oncology therapy. This study broadens the scope of bacteria as drug carriers. The constructed AISI strain specifically proliferated in tumors, with titers as high as 10^8^ CFU/g tissue, which is 1000 times higher than that in normal tissues. Incorporation of the QS system into *Salmonella* has been shown to initiate protein production only at high intratumoral bacterial titers.^[^ ^13a,c^ ^]^ Here, combined genomic modification of the strain provided an optimized QS system for rapid protein expression. This strategy does not rely on exogenous small molecules to enhance initiation sensitivity^[^ ^13b^ ^]^ and thus has great potential for application.

In the future, the controllable reduction in bacterial virulence described herein could serve as a modular strategy. Understanding the expression and regulation of virulence genes in *Salmonella* and other strains such as *Listeria monocytogenes*,^[^ ^2c^ ^]^ may allow for the application of similar approaches. Beyond their clear potential in cancer therapy, AISI strains could find utility in other clinical settings, such as tumor foci tracing and thrombolytic therapy,^[^ ^2a^ ^]^ and can provide a common platform for programmable interfaces in various environments. As our knowledge of bacterial virulence and antitumor mechanisms continues to expand, along with advancements in genetic circuit editing and regulation, the development of live microbe‐mediated antitumor therapeutics that balance safety and efficacy will accelerate the transformation and application of this biotherapy.

## 4 Experimental Section

### Cells and Bacteria

B16F10 mouse melanoma cells, H22 mouse hepatoma cell line, and RAW264.7 mouse leukemia macrophage cells were all maintained in the laboratory. The cells were cultured in Dulbecco's modified Eagle medium (DMEM) (Gibco) supplemented with 10% fetal bovine serum (FBS, Gibco) and a mycoplasma removal agent (Yeasen, 40607ES03, Shanghai, China). The attenuated *Salmonella typhimurium* VNP20009 and AHL indicator strain CV026 (the *cviI*‐deficient mutant of CV31532) were preserved in the laboratory. Strains were cultured using Luria–Bertani (LB) medium. Gene‐deficient VNP20009 strains were obtained with the aid of the pCas/pTAT dual‐plasmid system to knockout or replace specific genes in the bacterial genome. Multiple primers were designed based on the sequences flanking the targeted gene segment and the sequences of the regions not deleted at both ends of the knockout gene. PCR amplification was performed to screen strains with successful knockout/replacement of the target gene. Detailed information on the various defective strains can be found in Table S1 (Supporting Information).

### Plasmid Construction

Plasmids containing the J23100/sifB promoter for expressing target proteins and the sequence element Axe/Txe (AT) for preventing plasmid loss were stored in the laboratory. Additionally, the pTD103luxI plasmid, which includes the pLuxI promoter and positive feedback quorum‐sensing elements, was obtained from Addgene (https://www.addgene.org/). Some of the target genes have tag sequences added at the C‐terminus, including myc‐tags and his‐tags, for subsequent detection. All the plasmids used were constructed using the Uniclone One Step Seamless Cloning Kit (Genesand, SC612, Beijing, China) and were subsequently transformed into wild‐type VNP20009 or genetically defective VNP20009 competent cells via electrotransformation. The strain that was transformed into the empty plasmid was used as a control. Detailed information on the various engineered strains ultimately obtained based on the different plasmids can be found in Table S1 (Supporting Information).

### Biofilm Formation and Extracellular Polysaccharide Assays

For the measurement of biofilm content, the activated strains were subcultured at a 1:100 ratio into fresh medium and grown at 37 °C with shaking at 220 rpm until the OD_600_ reached 0.6–0.8. Then, the cultures were diluted 1:100 into glass tubes containing fresh M63 medium and incubated at 37 °C with gentle shaking at 100 rpm for 36 hours. Afterward, the tubes were washed twice with PBS, air‐dried, and stained with 2% crystal violet. The biofilms appeared as purple rings at the air‒liquid interface. The biofilm was quantitatively dissolved in 95% ethanol, after which the OD_570_ was measured.

For the quantification of extracellular polysaccharides in the strains, single clone was picked from the agar plate and incubated overnight at 37 °C. An equal volume of bacterial culture (OD_600_ = 1.0) was centrifuged and then resuspended in the bacterial extracellular polysaccharide extraction reagent (Solarbio, EX1750). The resuspension was incubated at 80 °C in a water bath for 6 hours. After centrifugation at 10000 × g, the supernatant was collected, which contained the extracellular polysaccharides. Based on the phenol‒sulfuric acid method, a color reaction was performed for each sample using a polysaccharide content assay kit (ZCIBIO, ZC‐S0885, Shanghai, China), the OD_490_ of each sample was measured, and the polysaccharide content was calculated based on the standard curve.

### EPS Visualization with TEM

The collection, staining and fixation of different bacterial samples were performed according to previous methods.^[^ ^8^ ^]^ In brief, the samples were fixed for 20 minutes on ice with 2.5% glutaraldehyde and 2% paraformaldehyde in osmotically adjusted buffer (0.1 M sodium cacodylate, 0.9 M sucrose, 10 mM CaCl_2_, 10 mM MgCl_2_) containing 0.075% ruthenium red and 75 mM lysine acetate. The samples were rinsed three times with osmotically adjusted buffer containing 0.075% ruthenium red before being fixed for 1–2 hours on ice with 1% osmium tetroxide in osmotically adjusted buffer containing 0.075% ruthenium red. The sample was first dehydrated using a graded series of ethanol (30%, 50%, 70%, 80%) for approximately 15 minutes at a time, followed by a graded series of acetone (90%, 95%) for approximately 15 minutes at a time. Finally, the strains were dehydrated twice with absolute acetone for 20 minutes each. The specimen was immersed in a 1:1 mixture of absolute acetone and the final Spurr resin mixture for 1 hour at room temperature before being moved to a 1:3 mixture of absolute acetone and the final resin mixture for 3 hours and overnight. The specimen was inserted in an Eppendorf tube containing Spurr resin and heated at 70 °C for more than 9 hours. The material was sectioned on a Leica EM UC7 ultratome, and the sections were stained with uranyl acetate and alkaline lead citrate for 5 to 10 minutes before being examined via a Hitachi HT‐7700.

### Bacterial Real‐Time Growth Curves and Fluorescence Intensity Assays

The growth curves and fluorescence expression curves of different attenuated *Salmonella* strains in LB medium or N‐minimal medium were monitored using a microplate reader (Biotek, Winooski, USA). The changes in the OD_600_ and expression of red fluorescent protein (RFP, excitation wavelength, 550 nm, emission wavelength, 585 nm) at 37 °C were continuously monitored for the different strains. The sensitivity of the quorum sensing system in bacteria was directly determined by the OD_600_ values at which different strains began expressing the fluorescent protein. A lower OD_600_ value corresponded to a higher sensitivity.

### Bacterial Intramacrophage Viability Assays

The assessment of bacterial intramacrophage viability was performed partially optimized following the methods described previously.^[^ ^4a^ ^]^ Briefly, RAW264.7 macrophages were seeded in a 12‐well plate and cultured continuously. Subsequently, the activated different strains were cocultured with the cells for 1 hour. Afterward, the cells were washed three times with PBS and cultured in medium containing 75 µg mL^−1^ gentamicin for another 1 hour or 5 hours to kill extracellular bacteria, after which the total number of surviving bacteria in the cell was detected. All cells from the plate were collected, washed with PBS, and then lysed with a PBS solution containing 0.5% Triton X‐100 (Sigma–Aldrich, 648 462, St. Louis, MO, USA) at room temperature. Finally, the lysed samples were serially diluted and plated on LB agar plates, followed by overnight incubation at 37 °C, after which the colony‐forming units on the plates were counted. The relative change of intracellular bacteria number after 5 hours of incubation versus 1 hour of incubation demonstrated the ability of the bacteria to survive and proliferate in macrophages.

### Neutrophil Activity Assay

To prepare peritoneal neutrophils, a solution consisting of 5% starch broth was created by dissolving 1.8% nutritional broth (Solarbio, N8300, Beijing, China) and 5% soluble starch in water. Subsequently, 1 mL of the prepared starch broth solution was intraperitoneally administered to eight‐week‐old female C57BL/6J mice. After 6 or 8 hours, peritoneal neutrophils were extracted. Neutrophils were subsequently cocultured with different bacteria in 96‐well plates. The free NETs were stained with the nucleic acid dye propidium iodide (PI), and the absorbance was measured in real time using a microplate reader. The supernatant was collected and subjected to reactive oxygen species (ROS) detection via mouse ROS ELISA kits (YIFEIXUE, YFXEM00485, Nanjing, China). After the mice were thoroughly sedated, the eyeball was removed, and blood anticoagulant was injected to obtain whole blood. A peripheral blood lymphocyte isolation kit (Solarbio, P8620) was used to collect peripheral blood lymphocytes. To detect active neutrophils in mouse peripheral blood, the cells were labeled with the following anti‐mouse antibodies: CD11b‐APC (BD, 553 312), Ly6G‐BV421 (BD, 562 737), and CD62L‐PE (BD, 553 151). After 30 minutes at 4 °C, the cells were washed 2–3 times with buffer to eliminate unbound antibodies before being analyzed on the instrument.

### Bacterial RNA‐Seq Analysis

All transcriptome data analysis and visualization were conducted using R (version 4.1.2). For key genes related to *Salmonella* survival within macrophages, the ClusterProfiler R package was used to perform Gene Ontology (GO) enrichment analysis on publicly available transcriptome data from Sofia Eriksson and colleagues.^[^ ^33^ ^]^ Specifically, genes that were significantly upregulated in *Salmonella* within macrophages at 4, 8, and 12 hours (log2FC > 1, p < 0.05) were subjected to GO enrichment analysis. The results of the enrichment analysis were visualized, which revealed changes in all the genes included in the top‐enriched term. RNA‐seq library preparation of the wild‐type and *htrA*‐deficient VNP strains was performed by Novogene (Novogene Corporation, Inc., China). The Limma R package was used to obtain the fold change in gene expression (log2‐fold change, Log2FC) and the adjusted p values (p.adjust). Genes with |Log2FC| > 1 and p.adjust < 0.05 were considered to be significantly differentially expressed genes. The changes in the regulation of genes by various transcription factors were analyzed based on the Prokaryotic Transcription Factor Database RegulonDB and the *Salmonella* gene regulation network database SalmonNet to identify the transcription factors most affected by HTRA. These analyses provide valuable insights into the regulatory mechanisms and genes involved in *Salmonella* survival within macrophages and the impact of *htrA* gene deficiency on the bacterial transcriptome.

### Protein Interaction Prediction and Visualization

Using the method described by the DeepMind team,^[^ ^34^ ^]^ protein‒protein interactions of HTRA, RCSA, and LON were predicted using the AlphaFold‐Multimer tool. Specifically, AlphaFold2 was used in the CentOS 7 environment, and complete genetic databases were downloaded as references. Multiple protein interaction predictions were run with the parameters max_template_date = 2023‐01‐01, model_preset = multimer, and db_preset = full_dbs. The input sequences for the predictions are listed in Table S2 (Supporting Information). The predicted output results were further analyzed using rank_0, specifically relaxed_model_4_multimer_v2_pred_3 for the HTRA + RCSA prediction and relaxed_model_3_multimer_v2_pred_0 for the RCSA + LON prediction.

The visualization of the prediction results was accomplished using ChimeraX, developed by UCSF.^[^ ^35^ ^]^ Additionally, the key amino acid residues involved in protein interactions were analyzed based on an obtained solvent‐accessible surface area ≥15 A^2. The predicted alignment error (PAE) corresponding to rank_0 was visualized, along with the electrostatic and hydrophobic properties of the key residues on the molecular surface.

### Coimmunoprecipitation (co‐IP) and Western Blot (WB) assays

The strains obtained under different conditions were cultured overnight. Next, the bacterial supernatant and precipitate were separated by centrifugation. The bacteria in the precipitate were resuspended in PBS and subjected to ultrasonic disruption at 300 W on ice for 15 min (5‐sec sonication followed by a 5‐sec interval). After this, the suspension was centrifuged at 13,000 rpm for 10 min, after which the total bacterial protein was collected from the supernatant. The trichloroacetic acid (TCA) protein precipitation method was used to collect total protein from the supernatant as previously described.^[^ ^4a^ ^]^ The concentration of the collected protein was determined with a BCA assay. The protein solutions were heated in a dry bath incubator after mixing with SDS loading buffer and then subjected to Western blot analysis. His‐tag antibodies (Sigma–Aldrich, SAB1305538) were used to validate HTRA expression. Anti‐TLR4 antibodies (Bioworld Tech, BS3489, Nanjing, China), anti‐MyD88 antibodies (Abclonal, A21905), anti‐IκBα antibodies (Abclonal, A24909), anti‐phosphorylated IκBα antibodies (Abclonal, AP0999) and anti‐tubulin antibodies (Abclonal, A12289) were used to detect TLR4 signalling pathway activation. Secondary anti‐mouse IgG antibodies (CST, 7076) were used for detection. Grayscale analysis was conducted using ImageJ. Plasmids were constructed to express RCSA‐Myc with a Myc‐tag (RCSA‐Myc) or HTRA with a His‐tag (HTRA‐His), as well as both RCSA and HTRA‐His. These plasmids were separately electroporated into different strains. After overnight culture, protein extraction and coimmunoprecipitation were carried out according to methods described in previous research.^[^ ^36^ ^]^ In brief, the bacterial precipitate was resuspended in 1.5 mg mL^−1^ lysozyme and incubated on ice for 30 min. Subsequently, Triton X‐100 (1%) was added, and the mixture was supplemented with proteinase inhibitor (1:200). The cell lysates were centrifuged at 14000 × g for 15 min at 4 °C. The supernatant was then collected, and a portion of the sample was used as input. The remaining sample was subjected to overnight coincubation at 4 °C with protein A/G agarose beads (Thermo Scientific, 20 423), His‐tag antibodies, or Myc‐tag antibodies (ABclonal, AE009). Then, 50 µL of elution buffer (0.1 M glycine, pH = 3.0) was added, and the mixture was incubated for 5 min. The tube was briefly centrifuged, and the supernatant was collected for subsequent target proteins detection by western blotting.

### Bacterial Protein‒Protein Interaction Assay

To validate protein–protein interactions, a NanoBiT tagging system based on interaction‐mediated bioluminescent signal production when the epitope tag SmBiT bound to its complementation partner LgBiT was used.^[^ ^37^ ^]^ For this purpose, two fragments, SmBiT and LgBiT, were fused to bait and prey proteins, respectively. The interaction of these two proteins in strains leads to bioluminescent signal production after the addition of the chromogenic substrate. The resulting plasmids are listed in Table S1 (Supporting Information). Protein–protein interactions were visualized by plating the transformed strains on LB plates, and bioluminescence was analyzed using a Tanon imaging system (Tanon‐1600, Tanon). The bioluminescent signals of the bacteria in LB liquid media were quantified using a microplate reader.

### Real‐Time Quantitative PCR Assays

The macrophage line RAW264.7 was induced to the M2‐type using IL4 protein (MedChemExpress, HY‐P70653, Shanghai, China). Total intracellular RNA was extracted using a total RNA isolation kit (Vazyme, RC112), and total cDNA was obtained using a reverse transcription kit (Thermo scientific, M16315) according to the manufacturer's instructions. The detailed real‐time PCR sequences of primers used are shown in Table S3 (Supporting Information). Quantitative real‐time PCR was performed in a StepOnePlus Real‐Time PCR system (Applied Biosystems, USA), and the AceQ qPCR SYBR Green Master Mix (Vazyme, Q221‐01) was used for gene amplification.

### Detection of AHL

CV026 is an AHL and violacein‐deficient mutant of *C. violaceum* CV31532, and a nanomolar concentration of exogenously added AHL (such as C6‐HSL) could restore violacein production in CV026. Thus, the purple pigment (violacein) produced by CV026 can be directly observed and measured for quantification of AHL signaling molecule levels in the medium. The double‐layer plate method was used to detect AHL production as previously described.^[^ ^38^ ^]^ Specifically, LB solid agar media plates containing CV026 (at a ratio of 10%) were prepared. The supernatants from the cultures of different strains were collected and concentrated to 10% of the original volume by heating. The obtained concentrate was added dropwise to the center of the solid media prepared above or mixed with liquid media supplemented with strain CV026. After incubation for an appropriate time, the AHL concentration in media from different modified strains was evaluated according to the degree to which violacein was produced in CV026.

### Animal Models

All animal experimental methods and protocols were approved by the Animal Care and Use Committee of Nanjing University (IACUC‐2003167) following the appropriate ethical guidelines. Female BALB/c mice (4–5 weeks old) and C57BL/6J mice (6–8 weeks old) were procured from Changzhou Cavens Animal Company. B16F10 cells (2 × 10^5^ cells per mouse) were subcutaneously injected into the right axillary region of C57BL/6J mice. A20 cells (1 × 10^6^ cells per mouse) or H22 cells (1 × 10^6^ cells per mouse) were subcutaneously injected into the right axillary region of BALB/c mice. When the tumor volume reached 80–120 mm^3^, different strains was intraperitoneally injected. Tumor measurements were conducted at regular intervals of 1–2 days using calipers. The tumor volume (v, mm^3^) was computed employing the formula v = a^2^b × 0.52, with “a” representing a smaller radius and “b” indicating a larger radius. Tumor growth over time for each mouse within each group was plotted by measuring tumor volume after specific labeling. Changes in the body weight of each mouse were recorded throughout the study. Survival curves were generated by daily monitoring of tumor‐bearing mice. Euthanasia was implemented when signs of adverse reactions such as pain, apathy, or tumor necrosis were observed or when humane endpoints were reached, defined as a tumor weight equal to 10% of the mouse's body weight. The anti‐CSF1R antibody (15 mg k g^−1^) (BioXCell, Clone: AFS98) was intraperitoneally administered twice every other day to remove macrophages from the body.

### Safety Evaluation

After administering the drugs to the tumor‐bearing mice, the animals were euthanized, and various organs (heart, liver, spleen, lung, and kidney) were dissected. Photographs were taken for visual observation to assess and compare any significant damage to the organs. Whole‐blood samples were collected from the tumor‐bearing mice via retroorbital puncture. The serum was separated and stored at −80 °C for subsequent biochemical tests, including the analysis of markers such as alanine transaminase (ALT) and aspartate transaminase (AST). Complete blood counts, blood biochemical analysis, histological examination of tumors and various organs via H&E staining, and fluorescence immunostaining of tumor paraffin sections were all conducted by Wuhan Servicebio Company.

### Bacterial Biodistribution

The activated different strains were injected into melanoma‐bearing mice. At various time points after administration, the mice were euthanized, and their tumors, hearts, livers, spleens, lungs, and kidneys were harvested. These tissues were minced and placed in a PBS solution containing 0.5% Triton X‐100. Complete homogenization was achieved using a tissue lyser (70 Hz, 60 s three times, with a 30 s interval). The supernatant obtained after homogenization was subsequently plated onto agar plates, which were cultured at 37 °C overnight. Bacterial colonies were counted, and the bacterial titers within each gram of tissue were calculated. The bacterial titer within the tumor was compared to the bacterial titers in different organs to assess the tumor‐targeting capability of different strains.

The activated different strains harboring the LuxCDABE plasmid were intraperitoneally injected into H22 hepatoma‐bearing mice at a concentration of 1 × 10^6^ CFU per mouse. At various time points, the colonization of the tumor site by these strains was monitored using an in vivo imaging system (PerkinElmer, IVIS Lumina III, Waltham, MA, USA). To more effectively visualize the fluorescence of bacteria within various organs of the mice, the mice were euthanized, their organs were dissected, and live imaging was conducted after administration.

### Enzyme‐Linked Immunosorbent Assay (ELISA)

After various administrations, the mice were bled to obtain serum and subsequently euthanized for tumor collection. The tumor was blended with tissue lysis reagent (Absin, abs9225, Shanghai, China) at a concentration of 10 mg tissue/50 µL lysis reagent and homogenized using a tissue lyser. Centrifugation was used to collect the supernatant. TNF‐α and IFN‐γ were detected in both the serum and tumor lysates using mouse TNF‐α ELISA kits (BYabscience, BY‐EM220852) and IFNγ ELISA kits (BYabscience, BY‐EM220140). IL‐6 and IL‐1β were detected in the liver and spleen tissue homogenate using mouse IL‐6 ELISA kits (BYabscience, BY‐EM220188) and IL‐1β ELISA kits (Lapuda, LA28804H).

### Flow Cytometry

Following euthanasia of tumor‐bearing mice, tumors were retrieved, minced and subsequently cultured in digestion medium (containing 10 U mL^−1^ collagenase I, 400 U mL^−1^ collagenase IV, and 30 U mL^−1^ DNase I, all diluted in HBSS) for 30 min. The mixture was then passed through a 40 µm cell filter to remove cell clumps, resulting in a single‐cell suspension. Afterward, an erythrocyte lysis solution (Solarbio, R1010) was added, and the suspension was kept on ice for 1–2 min to lyse the red blood cells. The cells were then washed with PBS. The cells were stained with a fixable viability dye (BD, 564 407) and incubated in the dark at room temperature for 15–20 min. Subsequently, staining was carried out using mouse‐specific antibodies, as described in Table S4. The cells were then fixed using a membrane‐fixing solution (BD, 565 388). To measure the bacterial load in the tissues, the weight of each organ was recorded, and 0.5% Triton X‐100 was added based on the weight (2 µL mg^−1^ of tissue). The tissues were homogenized using a tissue homogenizer and stored at 4 °C for 30 minutes. Following the removal of cell clumps with a cell strainer, bacteria exhibiting RFP fluorescence in the lysate were identified using a flow cytometer. Detection of strain‐induced apoptosis was performed in accordance with previous research.^[^ ^39^ ^]^ All analyses were conducted on a BD Canto II flow cytometer.

### Immunofluorescence Assays

B16F10 cells were inoculated into C57BL/6 mice in the right axillary region. The mice were intraperitoneally injected with different strains expressing RFP either constitutively or density‐regulated (1 × 10^6^ CFU per mouse) after tumor formation. The mice were euthanized, and the tumors were retrieved at different time points. After the tumors were embedded in optimal cutting temperature (OCT), tumor sections were obtained using a cryostat (Leica Biosystems, Wetzlar, Germany). These sections were stained with DAPI and observed under a fluorescence microscope (Carl Zeiss, Axioplan 2, Oberkohen, Germany) for imaging.

### Statistical Analysis

Data analysis was performed using GraphPad Prism, version 9.0 (GraphPad Software). For comparisons between two groups, Student's *t*‐test was used. When dealing with more than two groups, one‐way analysis of variance (ANOVA) was conducted, followed by Dunnett's multiple comparisons test. Schematic images were created using Adobe Illustrator. The data are reported as the mean ± SD. ns, no significance. *P < 0.05; **P < 0.01; ***P < 0.001; ****P < 0.0001.

## Conflict of Interest

The authors declare no conflict of interest.

## Supporting information

Supporting Information

## Data Availability

The data that support the findings of this study are available from the corresponding author upon reasonable request.
