# Supplementary material for: Programmable Bacteria with Dynamic Virulence Modulation System for Precision Antitumor Immunity
Source: Adv Sci (Weinh). 2024 Jul 26;11(36):2404069. doi: 10.1002/advs.202404069 (PMC11423194; doi:10.1002/advs.202404069)
Supplement: Supplementary file 1 — Supporting Information [file ADVS-11-2404069-s001.pdf]

ADVANCED SCIENCE
Open Access

Supporting Information

for *Adv. Sci.*, DOI 10.1002/advs.202404069

Programmable Bacteria with Dynamic Virulence Modulation System for Precision Antitumor Immunity

*Leyang Wu, Lin Li, Liyuan Qiao, Chenyang Li, Shuhui Zhang, Xingpeng Yin, Zengzheng Du, Ying Sun, Jiahui Qiu, Xiaoyao Chang, Bohao Wang and Zichun Hua**

Supporting Information

Programmable bacteria with dynamic virulence modulation system for precision antitumor immunity

Leyang Wu, *Lin Li*, *Liyuan Qiao*, *Chenyang Li*, *Shuhui Zhang*, *Xingpeng Yin*, *Zengzheng Du*, *Ying Sun*, *Jiahui Qiu*, *Xiaoyao Chang*, *Bohao Wang*, *Zichun Hua**

Leyang Wu and *Lin Li* contributed equally to this work.

L.Y. Wu, L. Li, L.Y. Qiao, C.Y. Li, S.H. Zhang, X.P. Yin, Z.Z. Du, Y. Sun, J.H. Qiu, X.Y. Chang, B.H Wang, Z.C. Hua
Department of Neurology of Nanjing Drum Tower Hospital and The State Key Laboratory of Pharmaceutical Biotechnology, School of Life Sciences and The Affiliated Hospital of Nanjing University Medical School, Nanjing University, Nanjing, 21008, Jiangsu, P. R. China
Email: zchua@nju.edu.cn

L.Y. Wu, Z.C. Hua
Nanjing Generecom Biotechnology Co., Ltd., Nanjing 210023, Jiangsu, P. R. China

L.Y. Wu, Z.C. Hua
Changzhou High-Tech Research Institute of Nanjing University and Jiangsu TargetPharma Laboratories, Inc., Changzhou 213164, Jiangsu, P. R. China.

Z.C. Hua
Faculty of Pharmaceutical Sciences, Xinxiang Medical University, Xinxiang 453002, Henan, P. R. China

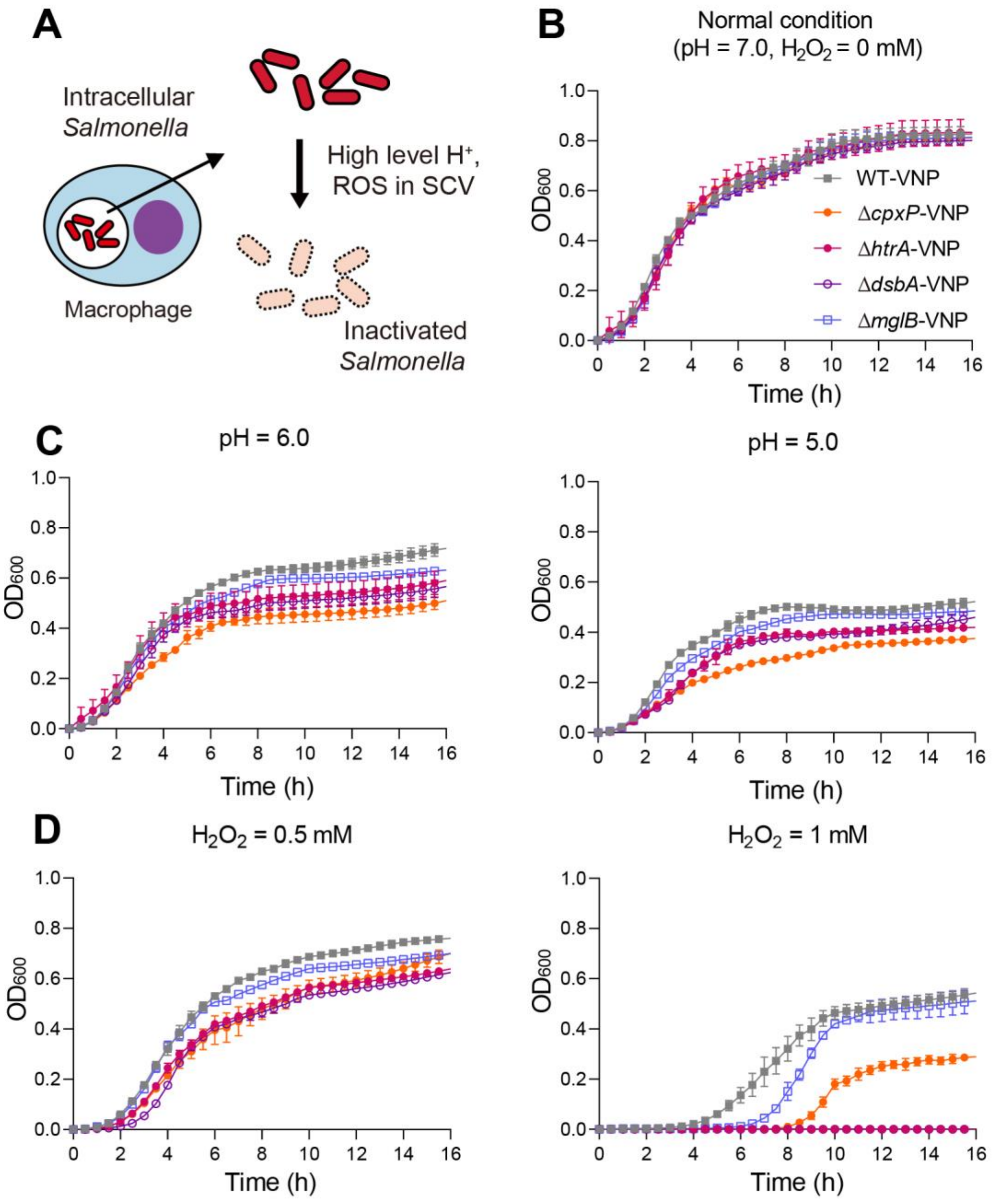

Figure S1. Comparison of acidic and oxidant resistance of five attenuated *Salmonella* strains. (**A**) Schematic of intracellular *Salmonella* killing by macrophages. Macrophages kill internalized intracellular bacteria by creating reactive oxygen species (ROS) and acidic stress. (**B**) Growth curves of five attenuated *Salmonella* strains (including WT-VNP, Δ*cpxP*-VNP, Δ*htrA*-VNP, Δ*dsbA*-VNP, and Δ*mlgB*-VNP) in liquid LB media under normal conditions (pH 7.0, H_2O_2 0 mM). (**C**) Growth curves of five attenuated *Salmonella* strains in liquid LB medium under acidic conditions at pH 6.0 (left) and pH 5.0 (right). (**D**) Growth curves of five attenuated *Salmonella* strains in liquid LB media supplemented with 0.5 mM H_2O_2 (left) or 1.0 mM H_2O_2 (right). *n* = 4 in B-D. All the growth curve data in B-D are relative values obtained by removing the OD600 values of the blank medium.

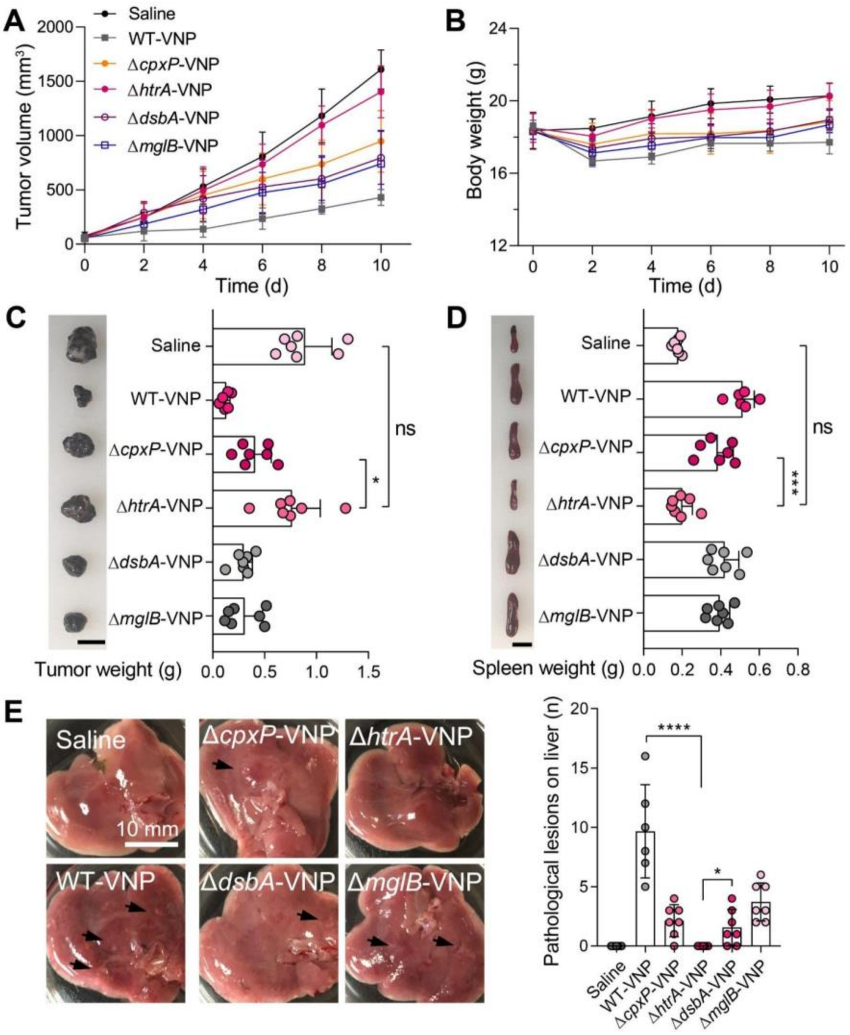

Figure S2. Comparison of antitumor effects and chronic toxicity of the five attenuated *Salmonella* strains.

(**A**) Assessment of the antitumor effects of the five attenuated *Salmonella* strains in a B16-F10 tumor-bearing mouse model. (**B**) Comparison of body weight changes after intraperitoneal administration of five attenuated *Salmonella* strains to tumor-bearing mice. The WT-VNP strain exhibited body weight loss after administration, but the Δ*htrA*-VNP strain did not exhibit significant body weight loss. (**C**) Comparison of tumor weights after 10 days of administration of five attenuated *Salmonella* strains. Representative tumor photographs (left) with comparative histograms of tumor weights (right) are shown. (**D**) Comparison of spleen weights after 10 days of administration of five attenuated *Salmonella* strains. Representative spleen photographs (left) with comparative histograms of spleen weights (right) are shown. (**E**) Comparison of liver injury after 10 days of administration of five attenuated *Salmonella* strains. Representative liver photographs (left) with statistical histograms of liver foci (right) are shown. The black arrows point to the liver foci. n = 6 or 7 per mouse in A-E. All error bars represent the s.d. Statistics were calculated using the two-tailed unpaired Student's t test with Welch's correction. ns, no significance. *, $P < 0.05$; **, $P < 0.01$; ***, $P < 0.001$.

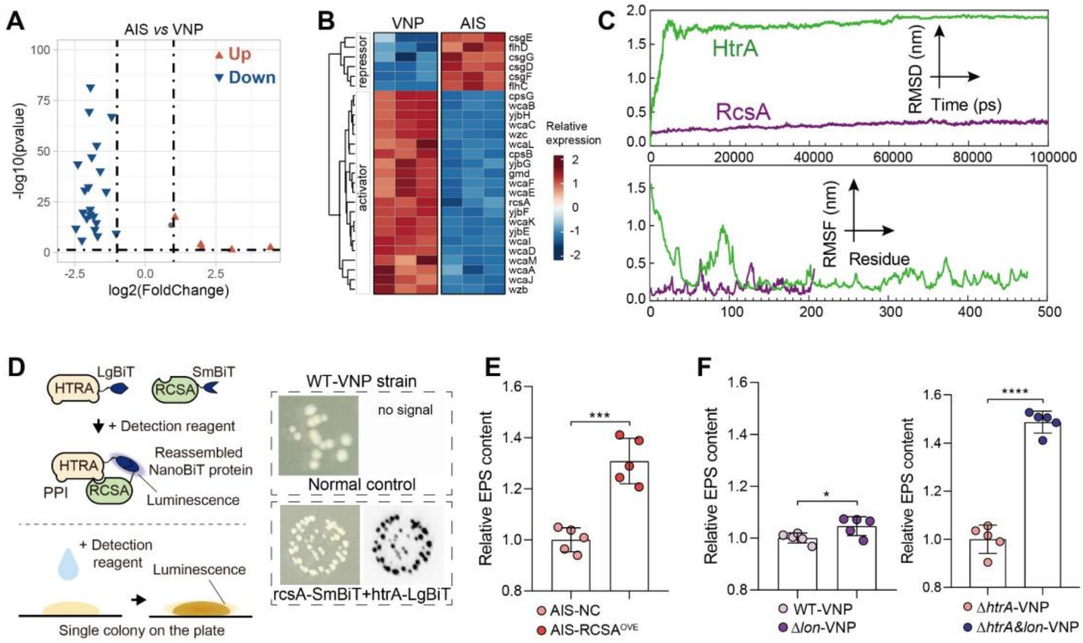

Figure S3. HTRA affects the bacterial extracellular polysaccharide content by competitively binding RCSA against LON.

(**A, B**) Compared to that in the VNP strain, the expression of the genes regulated by the RCSA transcription factor in the AIS strain is depicted in a volcano plot (A) and heatmap (B), where "activator" and "repressor", respectively, indicate the regulatory effects of RCSA on the gene, either activating or inhibiting transcription. (**C**) Dynamic PPI simulation assay with the molecular dynamic simulation tool GROMACS based on the initial file output from AlphaFold2 are shown. Over a 100,000 ps simulation period, the RMSD of HtrA rapidly stabilized after the initial relaxation phase, while the RMSD of RcsA remained consistent and stable, fluctuating within the 0.3 nm range. The RMSF of HtrA, excluding the relatively flexible N-terminal signal peptide (approximately 30 AAs), remains below 1 nm, and the RMSF of RcsA is maintained under 0.6 nm. (**D**) Schematic of the detection of protein interactions between HTRA and RCSA based on NanoBiT-mediated bioluminescence signaling (left). Representative colony bioluminescence images (right) are shown. (**E, F**) Comparison of the relative extracellular polysaccharide (EPS) content of different attenuated *Salmonella* strains. Overexpression of the *rcsA* gene (E) or defects in the *lon* gene (F) both significantly elevated the EPS content of the Δ*htrA*-VNP strain. All error bars represent the s.d. Statistics were calculated using the two-tailed unpaired Student's t test with Welch's correction. ns, no significance. *, $P < 0.05$; ***, $P < 0.001$; ****, $P < 0.0001$.

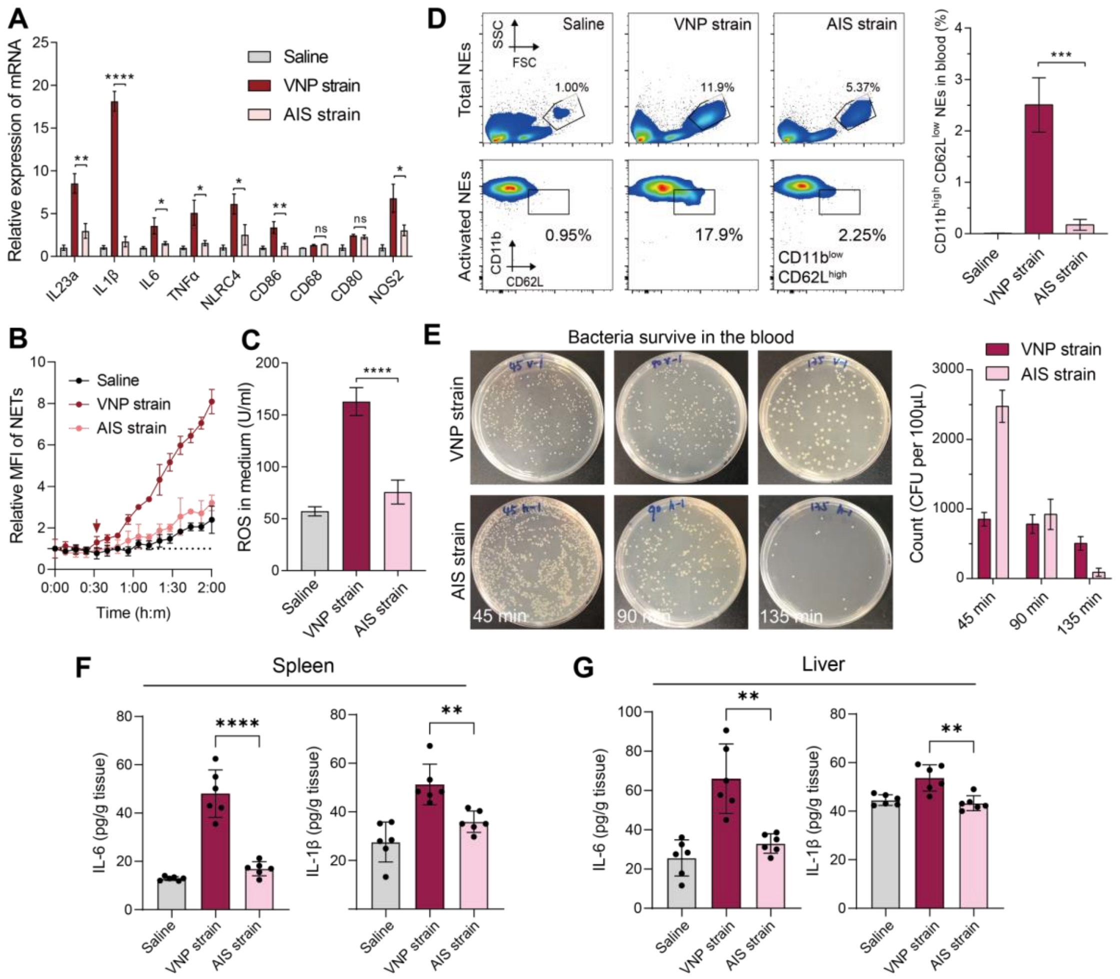

Figure S4. The bacterial immunogenicity of the AIS strain decreased.

(**A**) Detection of antitumor M1-type macrophage-related gene expression after coculturing different strains with M0-type macrophages for 6 hours (MOI 20) (n = 3). (**B**) Real-time monitoring of relative NET levels produced by neutrophils in the medium after different treatments (n = 3). (**C**) Comparison of ROS levels produced by neutrophils in the medium after different treatments at 90 min in (B) (n = 4). (**D**) Comparison of the percentage of activated neutrophils (CD11b^{high} CD62L^{low}) in peripheral blood after intravenous injection of VNP or AIS strains for 1 hour. Representative flow cytometric plots (left) and a bar graph corresponding to the counted activated neutrophils (right) are shown. (**E**) The bacterial titers in peripheral blood were examined at different time points (45/90/135 min) after intravenous injection of VNP and AIS strains. Representative photos of culture plates from one experiment (left) and a bar graph corresponding to the counted colony counts (right) are shown. (**F, G**) IL-6 and IL-1β levels in the spleen and liver of mice 1 day after administration with different strains (n = 6). All error bars represent the s.d. Statistics were calculated using the two-tailed unpaired Student's t test with Welch's correction. ns, no significance. *, P < 0.05; **, P < 0.01; ***, P < 0.001; ****, P < 0.0001.

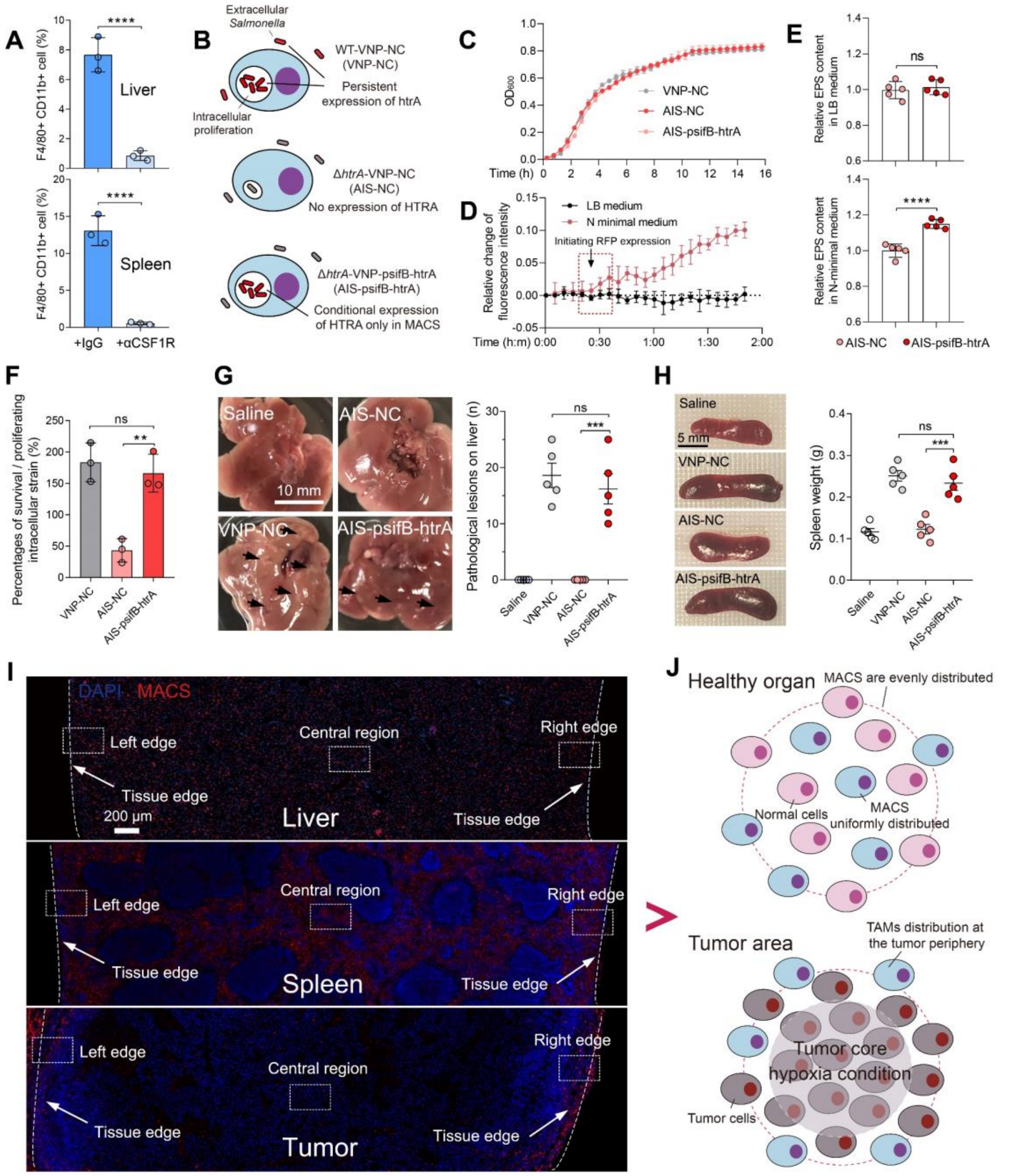

Figure S5. Macrophage-based strain clearance influences the *in vivo* distribution of AIS strains.

(**A**) Efficiency of macrophage clearance in the liver (top) and spleen (bottom) determined by macrophage-neutralizing antibodies (n = 3). (**B**) Schematic of the intramacrophage changes of three attenuated *Salmonella* strains (VNP-NC, AIS-NC, and AIS-psifB-htrA strains). (**C**) Comparison of growth curves of three attenuated *Salmonella* strains. (**D**) Real-time monitoring of RFP expression by AIS-psifB-RFP in N-minimal medium (simulating the environment inside macrophages). The promoter sifB initiated protein expression within 30 min. (**E**) Detection of the bacterial extracellular polysaccharide content of AIS-psifB-htrA strain after 12 h of incubation in LB medium or N-minimal medium (n = 5). (**F**) Comparison of the survival and replication ability of three attenuated *Salmonella* strains in macrophages (n = 3). (**G, H**) Comparison of hepatic lesion areas (G) and spleen weights (H) 1 day after intraperitoneal administration of saline or an

equal number of the three attenuated *Salmonella* strains ($n = 5$). (**I**) Immunofluorescence detection of macrophage distribution in the liver, spleen, and tumor. The white arrows point to the tissue margins. (**J**) Schematic of macrophage distribution in healthy organs and tumor regions. All error bars represent the s.d. Statistics were calculated using the two-tailed unpaired Student's t test with Welch's correction. ns, no significance. **, $P < 0.01$; ***, $P < 0.001$.

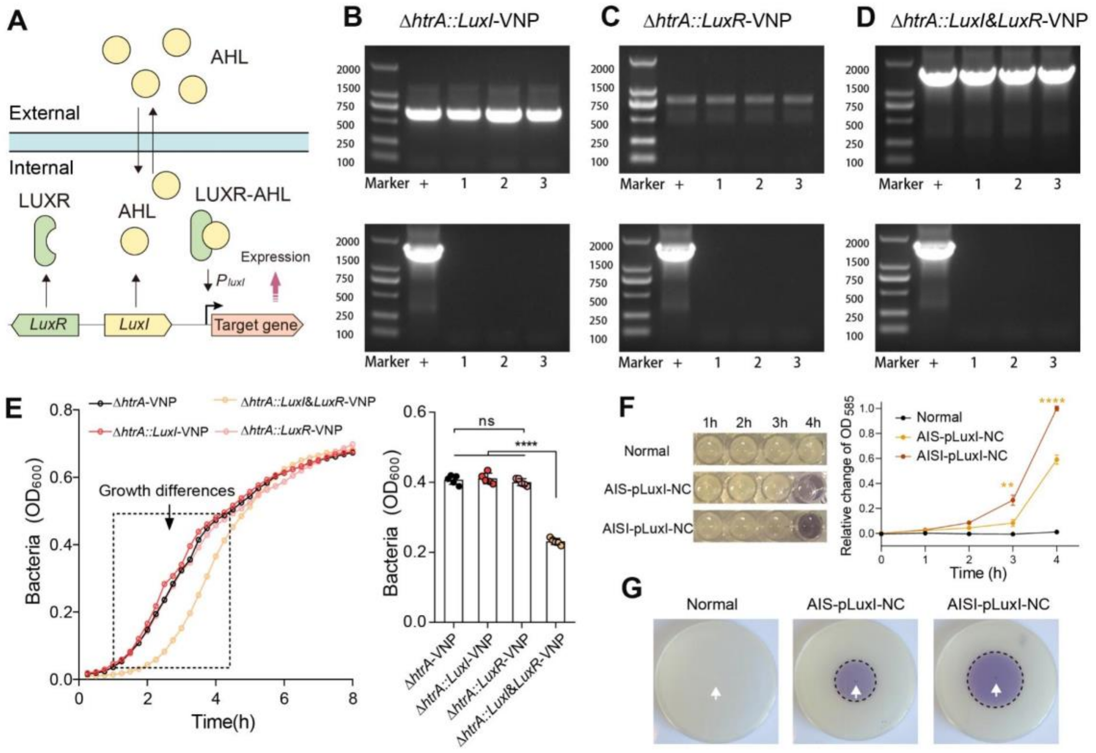

Figure S6. Validation and comparison of engineered strains obtained based on genome editing of the AIS strain.

(**A**) Schematic of the initiation principle of the QS system. The *LuxI* gene synthesizes AHL molecules, and AHL can bind to LUXR proteins produced by the *LuxR* gene, resulting in the LUXR-AHL complex, which can activate the LuxI promoter and turn on the expression of downstream genes. (**B-D**) Genomic validation of three defective strains, Δ*htrA::LuxI*-VNP (B), Δ*htrA::LuxR*-VNP (C) and Δ*htrA::LuxI&LuxR*-VNP (D). The PCR results for different strain genomes with inserted target genes (LuxI, LuxR, LuxI&LuxR) (top) and the knockout gene *htrA* (bottom) are shown. "+" shows the PCR results for the positive fragment of the inserted gene (top) and the genome of the wild-type VNP strain (bottom). Three monoclonal strains (1-3) were randomly selected for validation. (**E**) Comparison of the growth curves of the four attenuated *Salmonella* strains (left) and quantitative analysis of the OD_{600} of the strains at 3.5 hours (right). (**F**) Comparison of violacein formation after incubating strain CV026 with supernatants taken from different strains for 36 hours. Supernatants were collected from different strains after different incubation times (1-4 hours). Representative images of the wells (left) and the relative quantitative analysis (right) are shown. (**G**) Detection of AHL produced by different strains *via* violacein formation in the indicator strain CV026 based on a double-layer plate method. All error bars represent the s.d. Statistics were calculated using the two-tailed unpaired Student's t test with Welch's correction. ns, no significance. **, $P < 0.01$; ****, $P < 0.0001$.

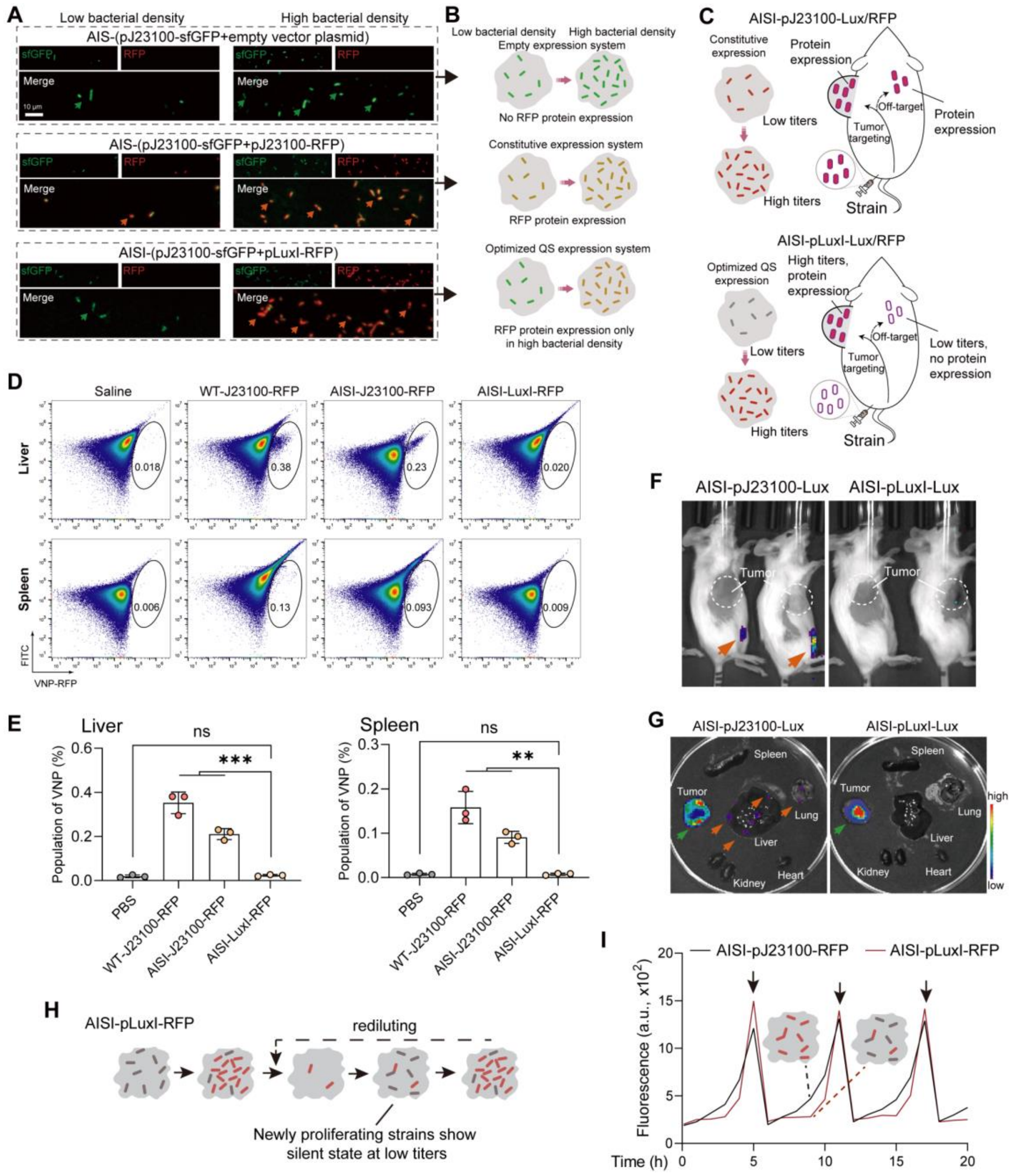

Figure S7. Assessment of the controllability of the optimized QS initiation system *in vivo* and *in vitro*.

(A) Observation of green fluorescence or red fluorescence production by three attenuated *Salmonella* strains *in vitro*. (**B**) Schematic of the fluorescence generation of the three engineered strains in (A). The strain transfected with the constitutively expressed sfGFP plasmid consistently produced green fluorescence. The strains transfected with constitutive sfGFP-expressing and RFP-expressing plasmids simultaneously and consistently produced red and green fluorescence, respectively. The strains transfected with constitutively expressed sfGFP and QS-initiated RFP plasmids consistently exhibited green fluorescence, with red fluorescence occurring only at high bacterial densities. Orange arrows point to strains producing both types of fluorescence. (**C**) Schematic attenuated *Salmonella* strains with two kind of expression systems (including AISI-pJ23100-Lux/RFP and AISI-pLuxI-Lux/RFP) *in vivo* showing bioenergetics after

intraperitoneal administration to tumor-bearing mice. The AISI-pJ23100-Lux/RFP strain sustained the production of bioluminescence or RFP so that light signals were detected at both enriched tumor sites and off-target sites. The AISI-pLuxI-Lux/RFP strain produces bioluminescence or RFP only within tumors while not at off-target sites. (**D, E**) Detection of the RFP-expressed VNP strain in the spleen and liver by flow cytometry on day 3 after administration. Representative flow cytometric plots (D) and statistical analyses histograms (E) are shown (n = 3). (**F**) Representative live imaging images of the two strains after 1 day of intraperitoneal administration. The circular dashed line shows the tumor site, and the orange arrows point to the site of administration. (**G**) Representative live imaging images of various tissues (including tumor, heart, liver, spleen, lung, and kidney) on day 3 after administration. The green arrows point to the biophotonic signal correctly expressed in the tumor tissue, and the orange arrows point to the biophotonic signal detected off-target to healthy organs. (**H**) Schematic of the dynamic activation of the AISI-pLuxI-RFP strain. The activated AISI-pLuxI-RFP strain (expressing RFP) was dilutied, and the de novo proliferating strain is in a silent state (no RFP expression), while it is only activated again after bacterial density reaching the QS initiation threshold. (**I**) The intensity of bacterial RFP fluorescence signals at different time points of AISI-pJ23100-RFP and AISI-pLuxI-RFP strains were detected as described in H. Black arrows indicate the dilution time points. All error bars represent the s.d. Statistics were calculated using the two-tailed unpaired Student's t test with Welch's correction. ns, no significance. **, $P < 0.01$; ***, $P < 0.001$.

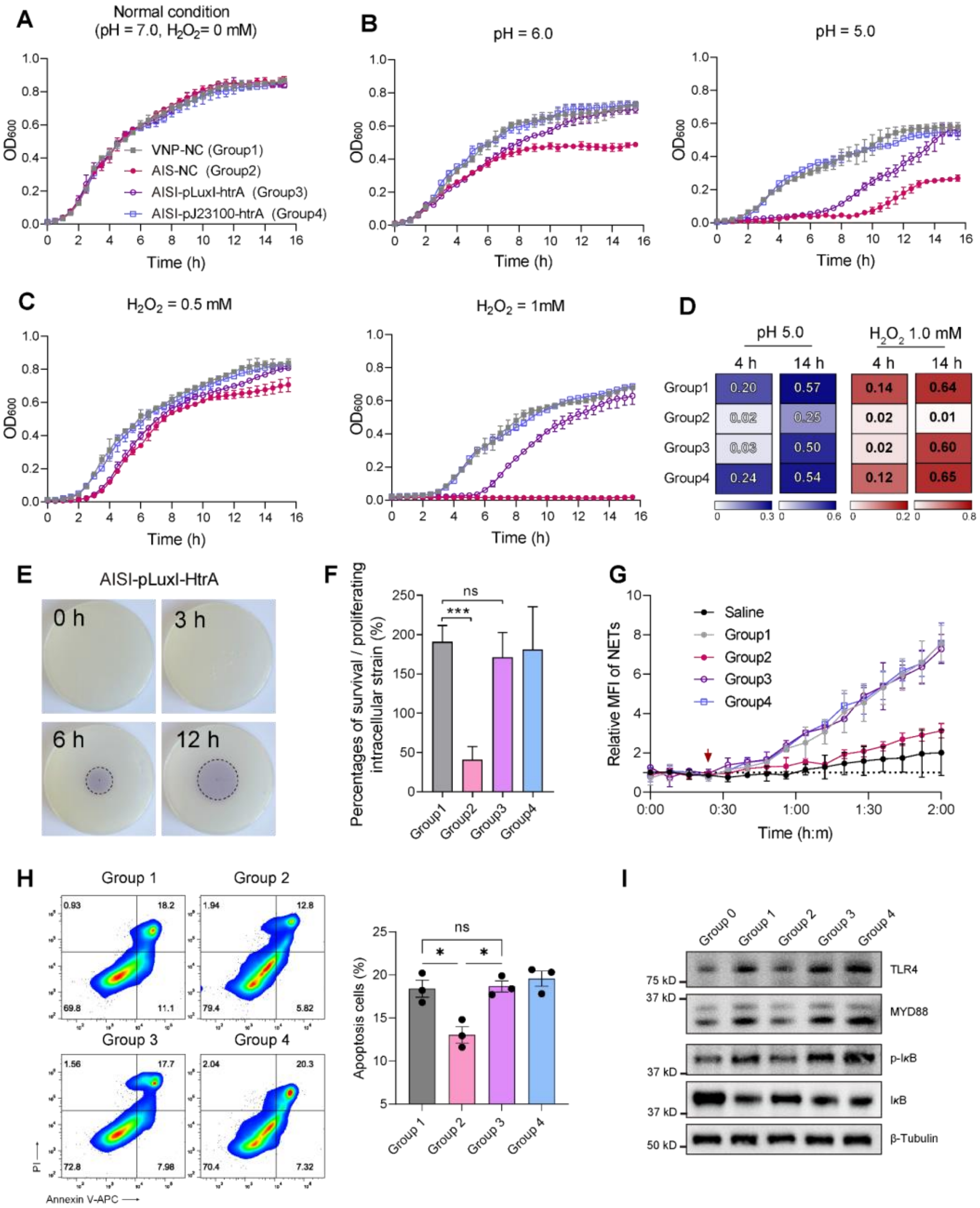

Figure S8. HTRA re-expression rescues the original biofunctional defects of attenuated *Salmonella* AIS.

(A) Growth curves of four attenuated *Salmonella* strains in liquid LB medium under normal conditions (pH 7.0, H_2O_2 0 mM) (n = 4). (**B**) Growth curves of four attenuated *Salmonella* strains in liquid LB medium under acidic conditions at pH 6.0 (left) and pH 5.0 (right) (n = 4). (**C**) Growth curves of four attenuated *Salmonella* strains in liquid LB medium supplemented with 0.5 mM H_2O_2 (left) or 1.0 mM H_2O_2 (right) (n = 4). (**D**) Comparison of the OD_{600} values of four attenuated *Salmonella* strains after 4 h of incubation in medium at pH 5.0 (left) or with the addition of 1 mM of hydrogen peroxide (right). The numbers displayed in the figure are the means (n = 3). (**E**) Detection of AHL produced by the AISI-pLuxI-htrA strain at different times *via* violacein formation in the indicator strain CV026 based on a double-layer plate method. (**F**) Comparison of the ability of four attenuated *Salmonella* strains to survive/proliferate in macrophages (n = 3). (**G**) Real-time monitoring of relative NET levels produced by neutrophils in the medium after different treatments (n = 3). (**H**) Representative flow cytometric plots (left) and

bar graphs comparisons (right) of B16F10 tumor cell apoptosis (PI+ Annexin V+) induced by the different strains (n = 3). (**I**) The activation status of TLR4-NF-κB signalling pathway was evaluated by western blots after 3 hours of co-incubation of different strains with macrophage RAW264.7. Significantly TLR4,MYD88 upregulation with activation of the NF-κB pathway was detected in the VNP-NC, AISI-pLuxI-htrA and AISI-pJ23100-htrA group. All error bars represent the s.d. All values of the growth curves in A-C are relative values obtained by removing the OD_{600} values of the blank medium. Statistics were calculated using the two-tailed unpaired Student's t test with Welch's correction. ns, no significance. ***, $P < 0.001$.

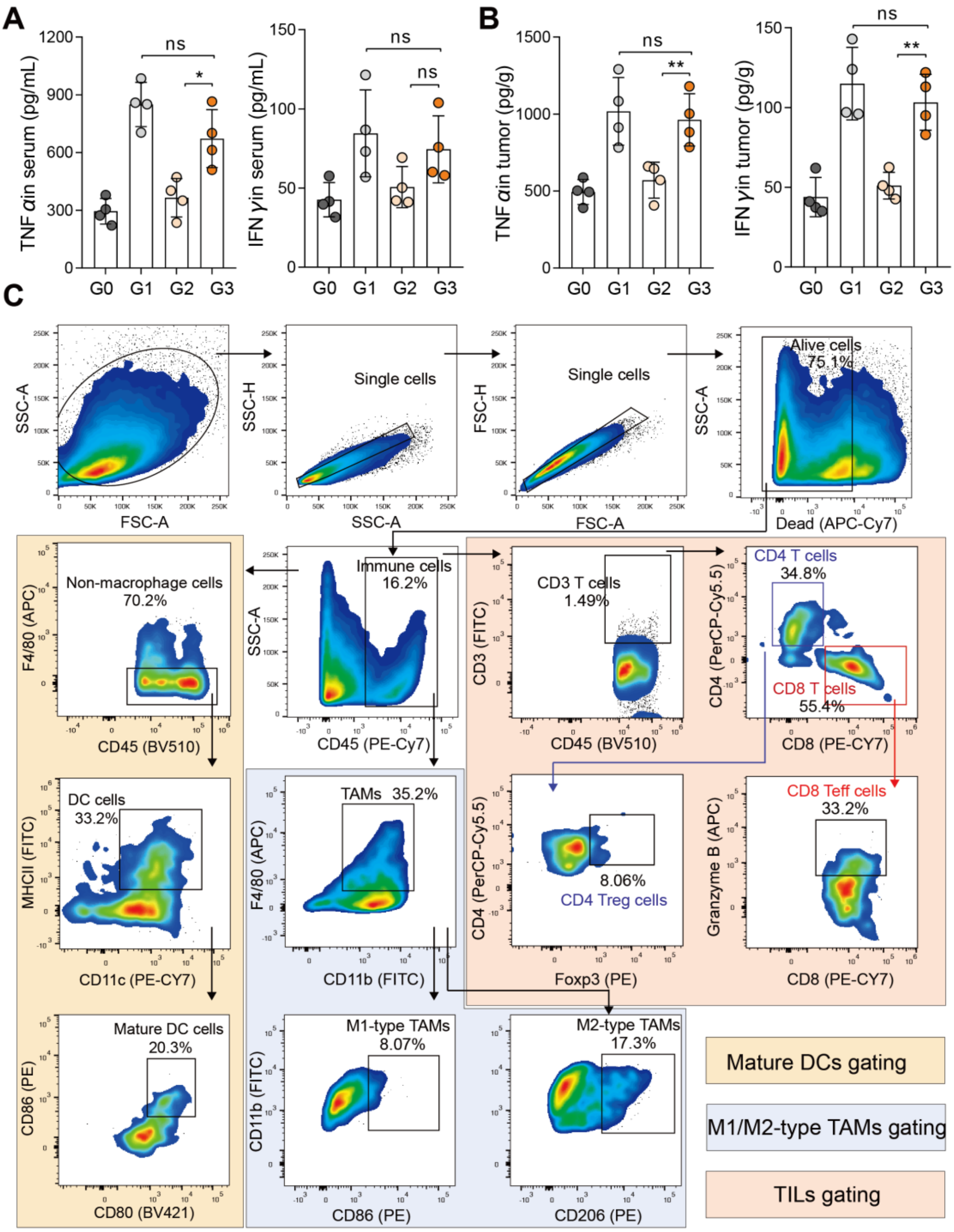

Figure S9. The novel engineered strain AISI-H effectively activates antitumor immunity.
(**A, B**) The concentrations of the antitumor cytokines TNFα (left) and IFNγ (right) were assayed in serum (A) and tumors (B). *n* = 4 mice per group. (**C**) Representative gating strategy for identifying dendritic cells (DCs) (yellow region), tumor-associated macrophages (TAMs) (blue region) and lymphocytes (orange region) with different phenotypes.

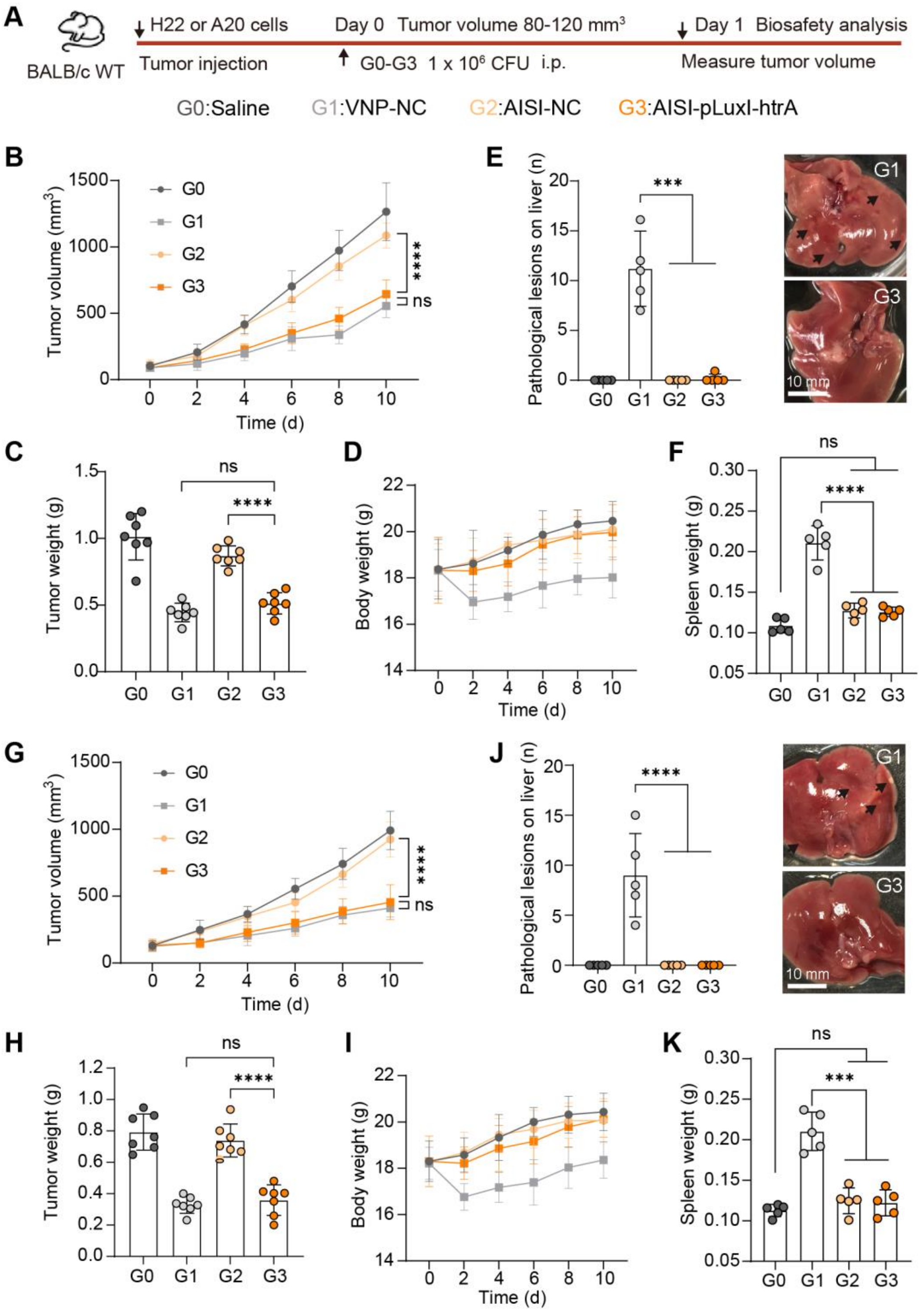

Figure S10. The novel engineered strain AISI-pLuxI-htrA shows robust anti-tumor efficacy and excellent safety profile in multiple tumor models.

(**A**) Schematic of the different strains used for antitumor therapy and biocompatibility analysis in subcutaneous H22 tumor model (**B-F**) or subcutaneous A20 tumor model (**G-K**). (**B**) Tumor growth profiles after different treatments in H22 tumor models (n = 7). (**C**) Tumors were weighed on day 10 after administration in B. (**D**) Changes in the body weights of tumor-bearing mice after different treatments in B. (**E**) Comparison of acute liver injury in each group of mice on day 1 after different administrations. Representative images of liver injury (left) with statistical comparisons of liver lesions number (right) are shown (n = 5). (**F**) Comparison of spleen weights 1 day after intraperitoneal administration of saline or an equal dose of the three attenuated Salmonella strains (n = 5). Scale bar = 5 mm. (**G-K**) Anti-tumour efficacy and safety evaluation of strains in the A20 subcutaneous tumour model, with experimental methods and time points consistent with those in B-F. Statistics were calculated using the two-tailed unpaired Student's t test with Welch's correction. All error bars represent the s.d. ***, $P < 0.001$; ****, $P < 0.0001$.

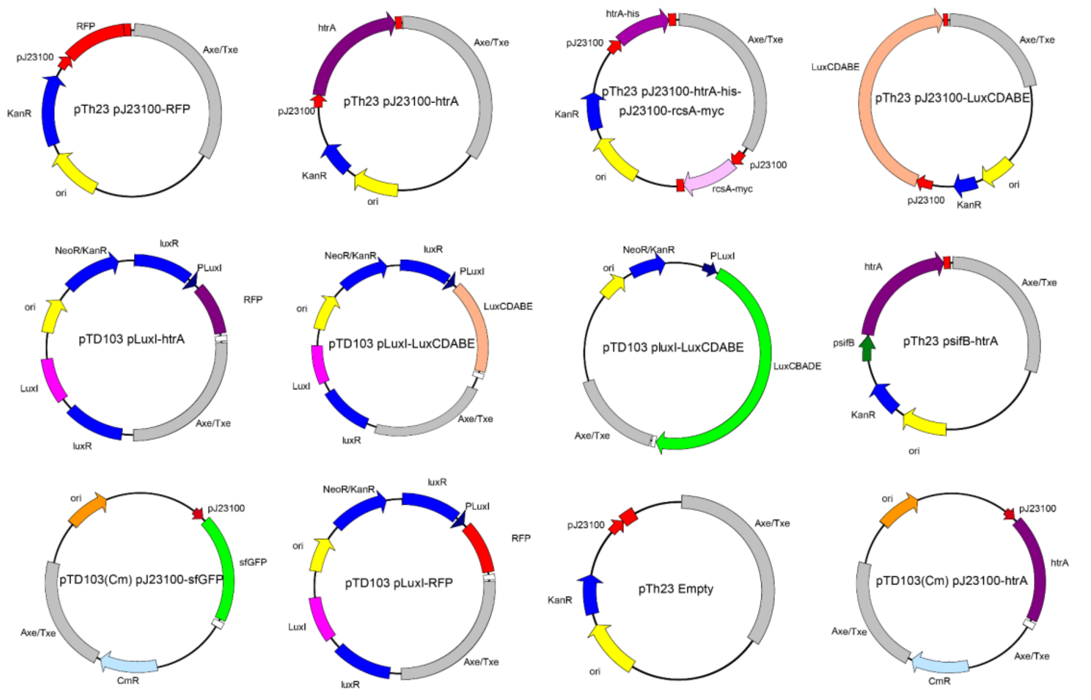

Figure S11. Representative plasmid profiles used in this study are shown.

Table S1. A list of strains and respective plasmids used in this study.

Strain #	Host Strain	Plasmid(s)	Description
**VNP**	VNP20009	N/A	Antitumor attenuated *Salmonella typhimurium*
**VNP-1**	VNP20009	pTh23 pJ23100-RFP	VNP strains constitutively expresses RFP
**VNP-2**	VNP20009	pTh23 pJ23100-htrA-his	VNP strains constitutively expresses HTRA -His
**VNP-3**	VNP20009	pTh23 pJ23100-rcsA-myc	VNP strains constitutively expresses RCSA-Myc
**VNP-4**	VNP20009	pTh23 pJ23100-htrA-his-pJ23100-rcsA-myc	VNP strains constitutively expresses HTRA-His and RCSA-Myc
**VNP-5**	VNP20009	pTD103(Cm) pJ23100-LuxCDABE + pTD103 empty	VNP strains constitutively expresses LuxCDABE
**VNP-6**	VNP20009	pTh23 pJ23100-lon-LgBiT- pJ23100-rcsA-SmBiT	VNP strains constitutively expresses LON-LgBiT

			and RCSA-SmBiT
**AIS**	Δ*htrA*-VNP20009	N/A	VNP strains defective in *htrA* gene
**AIS-1**	Δ*htrA*-VNP20009	pTh23 pJ23100-RFP	AIS strains constitutively expresses RFP
**AIS-2**	Δ*htrA*-VNP20009	pTD103 pLuxI-RFP	AIS strains expresses RFP in a QS-dependent manner
**AIS-3**	Δ*htrA*-VNP20009	pTD103 pLuxI-LuxCDABE	AIS strains expresses LuxCDABE in a QS-dependent manner
**AIS-4**	Δ*htrA*-VNP20009	pTD103(Cm) pJ23100-sfGFP + pTD103 pLuxI-RFP	AIS strains constitutively expresses sfGFP, and expresses RFP in a QS-dependent manner
**AIS-5**	Δ*htrA*-VNP20009	pTh23 psifB-htrA	AIS strains expresses HTRA with the aid of the sifB promoter
**AIS-6**	Δ*htrA*-VNP20009	pTh23 empty	AIS strains carrying empty plasmids
**AIS-7**	Δ*htrA*-VNP20009	pTh23 pJ23100-htrA-LgBiT- pJ23100-rcsA-SmBiT	AIS strains constitutively expresses HTRA-LgBiT and RCSA-SmBiT
**AIS-8**	Δ*htrA*-VNP20009	pTh23 pJ23100-Lon-LgBiT- pJ23100-rcsA-SmBiT	AIS strains constitutively expresses LON-LgBiT and RCSA-SmBiT
**AIS-9**	Δ*htrA*-VNP20009	pTh23 pJ23100-Lon-LgBiT- pJ23100-rcsA-SmBiT + pTD103(Cm) pJ23100-htrA	AIS strains constitutively expresses LON-LgBiT, RCSA-SmBiT and HTRA
**AISI**	Δ*htrA::LuxI*-VNP20009	N/A	Replacement of the *htrA* gene in AIS strain with the pJ23100-*LuxI* Seq.
**AISI-1**	Δ*htrA::LuxI*-VNP20009	pTD103 pLuxI-RFP	AISI strains expresses RFP in a QS-dependent manner
**AISI-2**	Δ*htrA::LuxI*-VNP20009	pTD103(Cm) pJ23100-sfGFP + pTD103 pLuxI-RFP	AISI strains constitutively expresses sfGFP, and expresses RFP in a QS-dependent manner
**AISI-3**	Δ*htrA::LuxI*-VNP20009	pTh23 empty	AISI strains carrying empty plasmids
**AISI-4**	Δ*htrA::LuxI*-VNP20009	pTD103 pLuxI-htrA	AISI strains expresses HTRA in a QS-dependent manner
**AISI-5**	Δ*htrA::LuxI*-VNP20009	pTh23 pJ23100-htrA	AISI strains constitutively expresses HTRA
**AISI-6**	Δ*htrA::LuxI*-VNP20009	pTD103 pLuxI-LuxCDABE	AISI strains expresses LuxCDABE in a QS-dependent manner
**AISI-7**	Δ*htrA::LuxI*-VNP20009	pTD103(Cm) pJ23100-LuxCDABE + pTD103 pLuxI-htrA	AISI strains constitutively expresses LuxCDABE, and expresses HTRA in a QS-dependent manner
**AISI-8**	Δ*htrA::LuxI*-VNP20009	pTD103(Cm) pJ23100-LuxCDABE + pTD103 empty	AISI strains constitutively expresses LuxCDABE
**AISR**	Δ*htrA::LuxR*-VNP20009	N/A	Replacement of the *htrA* gene in AIS strain with the pJ23100-*LuxR* Seq.
**AISR-1**	Δ*htrA::LuxR*-VNP20009	pTD103 pLuxI-RFP	AISI strains expresses RFP in a QS-dependent manner
**AISIR**	Δ*htrA::LuxI&LuxR*-VNP20009	N/A	Replacement of the *htrA* gene in AIS strain with the pJ23100-*LuxI*-pJ23100-*LuxR* Seq.
**Δ*rcsA*-1**	Δ*rcsA*-VNP20009	N/A	VNP strains defective in *rcsA* gene
**Δ*lon*-1**	Δ*lon*-VNP20009	N/A	VNP strains defective in *lon* gene
**Δ*lon*-2**	Δ*lon*-VNP20009	pTh23 pJ23100-htrA	Δ*lon*-1 strains constitutively express HTRA
**Δ*htrA&lon*-1**	Δ*htrA*-Δ*lon*-VNP20009	N/A	VNP strains defective in *htrA* and *lon* gene
**Δ*htrA&lon*-2**	Δ*htrA*-Δ*lon*-VNP20009	pTh23 pJ23100-htrA	Δ*htrA&lon*-1 constitutively expresses HTRA

**Δ*cpxP*-1**	Δ*cpxP*-VNP20009	N/A	VNP strains defective in *cpxP* gene
**Δ*dsbA*-1**	Δ*dsbA*-VNP20009	N/A	VNP strains defective in *dsbA* gene
**Δ*mglB*-1**	Δ*mglB*-VNP20009	N/A	VNP strains defective in *mglB* gene

Table S2. A list of protein sequences used for protein–protein interaction prediction in this study.

Protein	Amino acid sequence
**HTRA**	MKKTTLAMSALALSLGLALSPLSATAAETSSSAMTAQQMPSLAPMLEKVMPSVVSINVEGST<br>TVNTPRMPRNFQQFFGDDSPFCQDGSPFQNSPFCQGGGNGGNGGQQQKFMALGSGVII<br>DAAKGYVVTNNHVVDNASVIKVQLSDGRKFDAKVVGKDPRSDIALIQIQNPKNLTAIKLADS<br>DALRVGDYTVAIGNPFGLGETVTSGIVSALGRSGLNVENYENFIQTDAAINRGNSGGALVNLN<br>GELIGINTAILAPDGGNIGIGFAIPSNMVKNLTSQMVEYGQVKRGELGIMGTELNSELAKAMK<br>VDAQRGAFVSQVMPNSSAAKAGIKAGDVITSLNGKPISSFAALRAQVGTMPVGSKISLGLLRE<br>GKAITVNLELQQSSQSQVDSSTIFSGIEGAEMSNKGQDKGVVVSSVKANSPAAQIGLKKGDVI<br>IGANQQPVKNIAELRKILDSKPSVLALNIQRGDSSIYLLMQ
**RCSA**	MSTIIMDLCSYTRLGLSGYLVSRGVKKREINDIETVDELAIACGAHQPSVVFINEDCFIHTPSDS<br>QQIKQIINQHPDTLFIVFMAIANVHFDEYLLVRKNLLISSKSIKPDSLDTLLGDILKKESGISGTIN<br>LPTLSLSRTESSMLRMWMEGQGTIQISDRMNIKAKTVSSHKGNIKRKIKTHNKQVIYHVVRL<br>TDNVTNGIFVNMR
**LON**	MNPERSERIEIPVLPLRDVVVYPHMVIPLFVGREKSIRCLEAAMDHDKKIMLVAQKEASTDEP<br>GVNDLFTVGTVASILQMLKLPDGTVKVLVEGLQRARISALSDNGEHFSAKAEYLDSPAIDERE<br>QEVLVRTAISQFEGYIKLNKKIPPEVLTSLNSIDDPARLADTIAAHMPLKLADKQSVLEMSDVN<br>ERLEYLMAMMESEIDLLQVEKRIRNRVKKQMEKSQREYYLNEQMKAIQKELGEMDDAPDE<br>NEALKRKIDAAKMPKEAKEKAEAELQKLKMMSPMSAEATVVRGYIDWMVQVPWNARSK<br>VKKDLRQAQEILDTDHYGLERVKDRILEYLAVQSRVNKIKGPILCLVGPPGVGKTSLGQSIAKA<br>TGRKYIRMALGGVRDEAEIRGHRRTYIGSMPGKLIQKMAKVGVKNPLFLLDEIDKMSSDMR<br>GDPASALLEVLDPEQNVAFSDHYLEVDYDLSDVMFVATSNSMNIPAPLLDRMEVIRLSGYTE<br>DEKLNIAKRHLLPKQIERNALKKGELTVDDSAIIGIIRYYTREAGVRSLEREISKLCRKAVKQLLLD<br>KSLKHIEINGDNLHDYLGVQRFDYGRADSENRVGQVTGLAWTEVGGDLLTIETACVPGKGKL<br>TYTGSLGEVMQESIQAALTVVRARAEKLGINPDFYEKRDIHVHVPEGATPKDGPSAGIAMCT<br>ALVSCLTGNPVRADVAMTGEITLRGQVLPIGGLKEKLLAAHRGGIKTVLIPFENKRDLEEIPDN<br>VIADLDIHPVKRIEEVLTLALQNEPSGMQVVTAK

Table S3. A list of primers sequences used for RT–PCR in this study.

Primer	Forward (5'-3')	Reverse (5'-3')
**β-actin**	GCA CCA CAC CTT CTA CAA TGA G	TTG GCA TAG AGG TCT TTA CGG A
**IL1β**	GCA ACT GTT CCT GAA CTC AAC T	ATC TTT TGG GGT CCG TCA ACT
**TNFα**	CCC TCA CAC TCA GAT CAT CTT CT	GCT ACG ACG TGG GCT ACA G
**IL23α**	AAT AAT GTG CCC CGT ATC CAG T	GCT CCC CTT TGA AGA TGT CAG
**IL6**	TAG TCC TTC CTA CCC CAA TTT CC	TTG GTC CTT AGC CAC TCC TTC
**NLRC4**	ATC GTC ATC ACC GTG TGG AG	GCC AGA CTC GCC TTC AAT CA

**NOS2**	GTT CTC AGC CCA ACA ATA CAA GA	GTG GAC GGG TCG ATG TCA C
**CD68**	TGT CTG ATC TTG CTA GGA CCG	GAG AGT AAC GGC CTT TTT GTG A
**CD80**	TGC TGC TGA TTC GTC TTT CAC	GAG GAG AGT TGT AAC GGC AAG
**CD86**	TGT TTC CGT GGA GAC GCA AG	TTG AGC CTT TGT AAA TGG GCA
**MCP1**	TTA AAA ACC TGG ATC GGA ACC AA	GCA TTA GCT TCA GAT TTA CGG GT
**RANTES**	GCT GCT TTG CCT ACC TCT CC	TCG AGT GAC AAA CAC GAC TGC
**ARG1**	GAT TGG CAA GGT GAT GGA AG	TCA GTC CCT GGC TTA TGG TT
**FIZZ1**	GAA ATG CCA CCT TTT GAC AGT G	TGG ATG CTC TCA TCA GGA CAG
**IL10**	CCT GGA TCT GTA TCA CCG AAG C	CTC CGA CCA CTC TGC CTT GTT A
**TGFβ**	CTC CCG TGG CTT CTA GTG C	GCC TTA GTT TGG ACA GGA TCT G

Table S4. A list of antibodies for flow cytometric analysis used in this study.

Antibody	Fluorescence	Supplier	Catalog number
**CD45**	PE-Cy7	BD Biosciences	552848
**CD45**	BV510	BD Biosciences	740131
**CD62L**	PE	BD Biosciences	561918
**CD11b**	FITC	BD Biosciences	561688
**CD11b**	APC	BD Biosciences	561690
**F4/80**	APC	BD Biosciences	566787
**Ly6G**	PerCP-Cy5.5	BD Biosciences	560602
**Ly6G**	BV421	BD Biosciences	562737
**CD11c**	PE-Cy7	BD Biosciences	561022
**CD80**	BV421	BD Biosciences	566285
**CD3**	FITC	BD Biosciences	561798
**MHCII**	FITC	BD Biosciences	562009
**CD4**	PerCP-Cy5.5	BD Biosciences	561115
**CD8**	PE-Cy7	BD Biosciences	561097
**Foxp3**	PE	BD Biosciences	560414
**CD206**	APC	Invitrogen	17-2061-82
**CD206**	PE	Invitrogen	12-2061-82
**CD86**	PE	Invitrogen	12-0862-82
**GzmB**	APC	Invitrogen	17-8898-82

Source Data of Western Blots and Gels

Figure2J

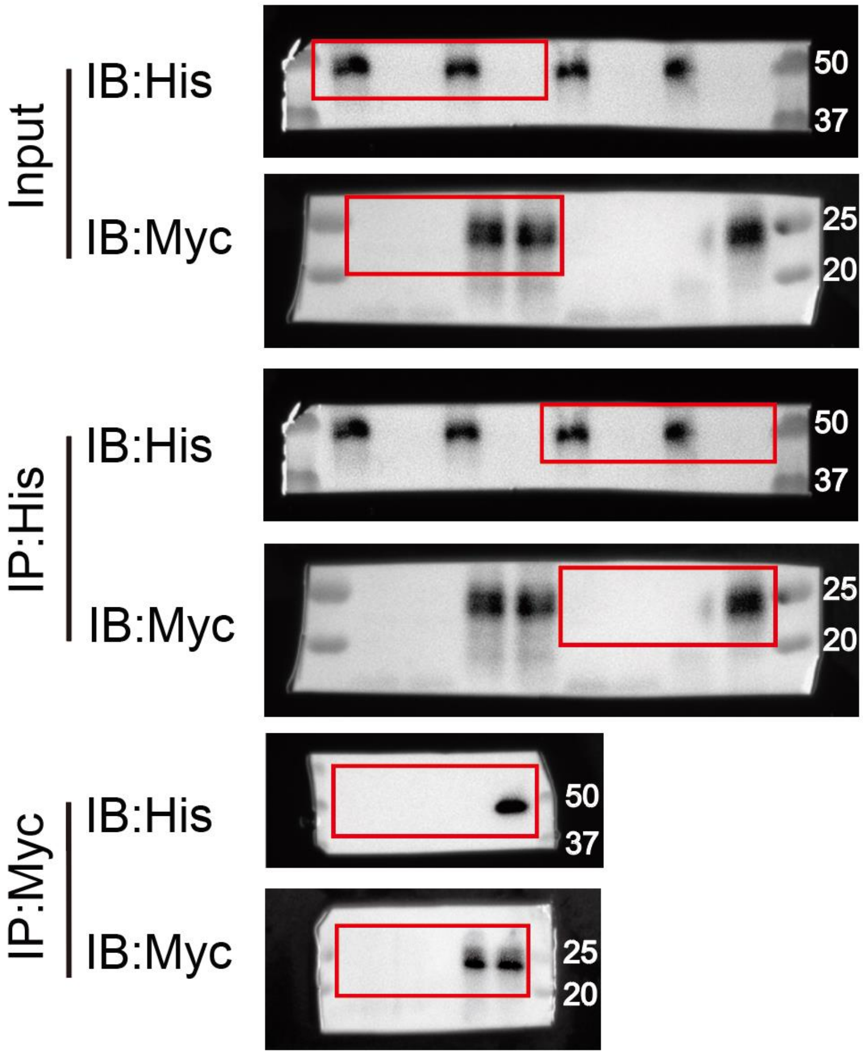

Figure4C

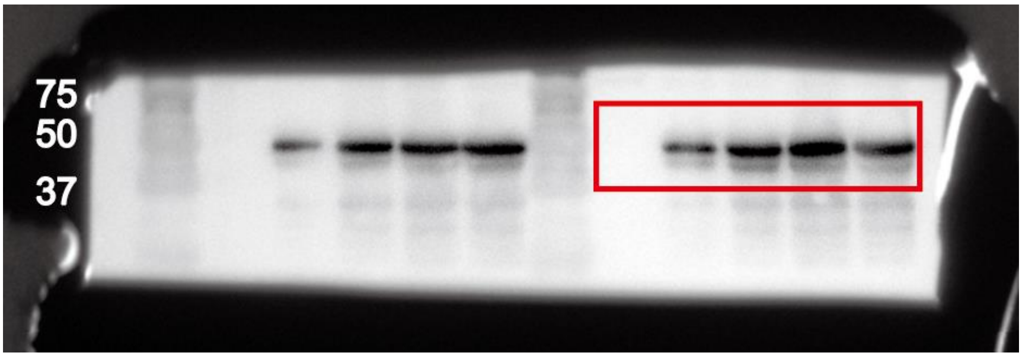

FigureS6B-D

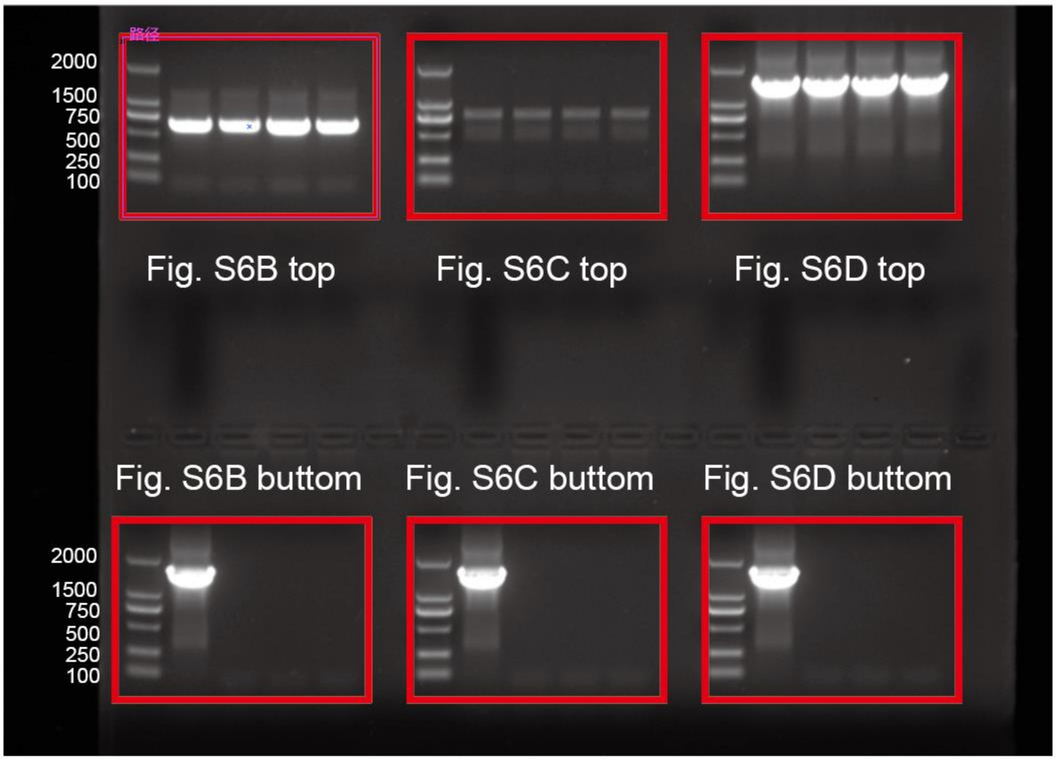

路径
2000
1500
750
500
250
100
Fig. S6B top
Fig. S6C top
Fig. S6D top
Fig. S6B buttom
Fig. S6C buttom
Fig. S6D buttom
2000
1500
750
500
250
100
